# EGR1 Nuclear Condensates Promote Renal Cyst Development in Polycystic Kidney Disease

**DOI:** 10.1002/EXP.20240285

**Published:** 2025-12-26

**Authors:** Chaoqun Ren, Zhaoxu Wu, Min Li, Yaqin Du, Hong Zhou, Yazhu Quan, Mengyao Xiong, Yiming Wang, Zhiwei Qiu, Shuai Zhu, Xiaowei Li, Jinzhao He, Cai Gao, Hui Cai, Tingting Li, Baoxue Yang

**Affiliations:** ^1^ State Key Laboratory of Vascular Homeostasis and Remodeling Department of Pharmacology School of Basic Medical Sciences Peking University Beijing China; ^2^ Department of Biomedical Informatics School of Basic Medical Sciences Peking University Beijing China; ^3^ Renal Division Department of Medicine Emory University School of Medicine and Nephrology Section Atlanta Georgia USA

**Keywords:** ADPKD, EGR1, cyst development, condensate, phase separation

## Abstract

Autosomal dominant polycystic kidney disease (ADPKD) is marked by aberrant cell proliferation driven by cAMP‐PKA and MAPK signaling pathways. EGR1, a transcription factor directly activated by the above two pathways, is critical in the over‐proliferation of tumor cells, which share similarities with cystic epithelial cells in ADPKD. This study utilized in vitro cell models, three‐dimensional (3D) cyst model, embryonic renal cystic model, and PKD mouse model to clarify the role of EGR1 in cyst development of ADPKD. We found the high expression and nuclear condensates of EGR1 in human ADPKD renal cyst epithelial cells and PKD mouse kidney tissue. Pharmacological inhibition of EGR1 retarded cyst enlargement in in vitro, ex vivo, and in vivo ADPKD models. Furthermore, EGR1 formed nuclear condensates with YAP1 and CBP via phase separation, leading to EGR1‐specific transcriptional activation and upregulation of cell‐cycle‐related genes (e.g., CCND1, CCNE1, and CDK4/6), thus promoting abnormal renal cystic epithelial cell proliferation. Disruption of EGR1 phase separation significantly alleviated cyst growth in the forskolin‐induced 3D spheroid model of mIMCD3 cells and MDCK cyst model. These findings demonstrate that phase separation‐mediated EGR1 condensates facilitate renal cyst development in ADPKD.

## Graphical abstract

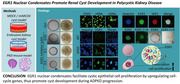

**Abbreviations**
1,6‐HD: 1,6‐hexanediol
3D: three‐dimensional
ADPKD: autosomal dominant polycystic kidney disease
cAMP‐PKA: cyclic adenosine monophosphate‐protein kinase A
CBP: CREB‐binding protein
EGR1: early growth response gene 1
ESRD: end‐stage renal disease
FSK: forskolin
GEO: gene expression omnibus
IDRs: intrinsically disordered regions
MAPK: mitogen‐activated protein kinase
PC1: polycystin 1
PC2: polycystin 2
PKD: polycystic kidney disease
YAP1: yes‐associated protein 1

## 1 Introduction

Autosomal dominant polycystic kidney disease (ADPKD) ranks as a prevalent genetic disorder affecting the kidneys, with global incidence rates ranging from 1 in 2500 to 1 in 1000. It stands as the fourth most common cause of end‐stage renal disease (ESRD) [1, 2]. Characterized by the continual growth of multiple fluid‐filled cysts within the kidneys, ADPKD progressively impairs renal function. This condition is responsible for 5%–10% of kidney failure, and approximately 50% of ADPKD patients end up with ESRD after the age of 60, imposing a serious burden on patients and society [3, 4]. However, there is still no effective therapy for curing ADPKD other than dialysis and kidney transplantation [5, 6, 7].

The majority of ADPKD cases are linked to genetic alterations in either the *Pkd1* (85%) or *Pkd2* (15%) genes, impacting the functionality of polycystin 1 (PC1) or PC2, respectively. These mutations lead to an impaired regulation of intracellular calcium levels [8] and trigger the activation of the PKA signaling cascade [9, 10, 11]. The misregulation of the cAMP‐PKA pathway is a pivotal element in ADPKD's development [12], especially the activation of downstream proliferation pathways such as mTOR [13], MAPK pathways, Wnt [14], and EGFR pathways [7]. It has been found that the abnormal Ras/MAPK pathway is mainly involved in cAMP‐stimulated human renal cyst cell proliferation [15]. However, there are gaps between signaling pathways mentioned above and initial cell proliferation in the mechanism of renal cyst development.

Early growth response gene 1 (EGR1), a crucial nuclear transcription factor (TF), orchestrates the regulation of numerous genes pivotal for cell proliferation [16, 17, 18, 19]. This factor is intricately linked to the onset and advancement of tumors, influencing tumor cell proliferation, invasion, and migration [16, 20, 21, 22, 23]. Conversely, EGR1 can also function as a transcriptional repressor by associating with specific transcription factors or co‐repressors, inhibiting the expression of certain genes and thus regulating processes such as cell differentiation and apoptosis [24]. In addition to its transcriptional regulatory roles, EGR1 is a key factor in extracellular matrix (ECM) production and stress responses. It modulates the expression of various matrix metalloproteinases (MMPs), and its expression can be induced by a wide range of stress signals, such as endoplasmic reticulum stress and oxidative stress.

Notably, activation of EGR1 by the MAPK pathway leads to its association with the cyclin D1 promoter, thereby augmenting cyclin D1 expression. This interaction facilitates the transition of cells from the G1 to S phase, facilitating cell proliferation [18, 25]. Moreover, EGR1 boosts tumor proliferation by elevating other cell cycle proteins, including cyclin D2 and CDK4 [26, 27, 28]. The activation pathways for EGR1—cAMP‐PKA, MAPK, and EGF/EGFR—are also significant in the development of ADPKD, underscoring the relevance of these pathways [29, 30, 31, 32]. Besides, it has been demonstrated that EGR1 regulates the transcription of the *Pkd1* gene, which encodes polycystin‐1, a protein crucial for maintaining normal renal function and preventing cyst formation [33]. Considering the extensive pro‐proliferative effect of EGR1, particularly in the complex regulation of the cell cycle, we hypothesize that it may possess significant potentials in driving the proliferation of cystic epithelial cells, thus contributing to renal cyst formation in ADPKD.

Here, we utilized distinct in vitro, ex vivo, and in vivo experimental models of ADPKD to reveal the biological effect and mode of action of EGR1 responding to cAMP‐PKA signal pathway in renal cyst development. Our experimental results provide proof of concept that EGR1 condensate is an important regulatory factor in ADPKD progression.

## 2 EXPERIMENTAL DETAILS

### 2.1 Mouse Model

Kidney‐specific *Pkd1* knockout mice were developed by breeding *Pkd1* ^flox/flox^ mice on a C57BL/6 genetic background. These mice were produced by crossing Pkd1^f^ ^lox/+^ mice, originally obtained from Dr. Stefan Somlo's lab, with *Ksp*‐Cre transgenic mice sourced from Dr. Peter Igarashi's lab, as has been detailed previously [15, 34]. Genotyping was carried out at birth (postnatal day 0), and the mice were subsequently euthanized on postnatal day 5. All animal experiments adhered to the ethical guidelines granted by the Peking University Health Science Center Committee on Animal Research.

### 2.2 MDCK Cyst Model

MDCK cells were prepared in a type I collagen (Sigma) solution mixed with modified Eagle's medium and 10 mM HEPES at pH 7.2. The cell mixture was then distributed into 24‐well plates and maintained in DMEM/F12 medium enriched with 10% FBS. To induce cyst formation, 10 µM forskolin (FSK, Sigma) was added from day 0. The culture medium was refreshed every 12h. Cyst growth was monitored by photographing the cultures (30–40 cysts per well) every 2 days starting from day 4. The cysts' diameters were measured utilizing Image J NIH software (NIH Image, Bethesda, MD).

### 2.3 Embryonic Kidney Cyst Model

Embryonic kidneys from ICR mice at day 13.5 of gestation were harvested and cultivated in transwells (Corning) employing a protocol outlined in earlier studies [15, 32]. The culture medium was DMEM/F12, enriched with 2 mM L‐glutamine, 250 U mL^−1^ penicillin, 250 µg mL^−1^ streptomycin, 10 mM HEPES, and a growth supplement consisting of 5 µg mL^−1^ each of insulin and transferrin, 2.8 nM selenium, 25 ng mL^−1^ prostaglandin E, 32 pg mL^−1^ T3, and 100 µM 8‐Br‐cAMP (Sigma) to stimulate cyst formation. ML264 was added from day 0 with medium refreshment occurring every 12 h. Over a 6‐day culture period, significant cyst enlargement was observed in response to the 8‐Br‐cAMP stimulation. The development and growth of these cysts were documented through micrographs taken on days 0, 2, 4, and 6 using a Nikon Ti2‐U microscope. Analysis of cystic and total kidney areas was done utilizing Image J NIH software (NIH Image, Bethesda, MD).

### 2.4 Production and Purification of Recombinant EGR1 Proteins

For the production of recombinant EGR1 proteins, plasmids containing cDNA sequences encoding these proteins were introduced into *Escherichia coli* BL21 (DE3) strains (Transgen) using chemical transformation, under ampicillin selection. The transformed *E. coli* BL21 cells were propagated in TB liquid medium (Solarbio) and cultivated at 37°C until reaching an optical density of 0.6 to 0.8. Protein expression was then induced through adding 0.2 mM isopropyl β‐D‐1‐thiogalactopyranoside and maintaining the culture at 16°C for 16 h. Following this, cells were gathered via centrifugation, and cellular disruption was gained through sonication in a lysis buffer (50 mM Tris‐HCl, 500 mM NaCl, pH 7.5). The cell debris was removed by centrifugation at 14 000*g* for 30 min, and protease inhibitors (Roche) were supplemented to the supernatant to prevent protein degradation. The clear lysate was subjected to initial purification using Ni‐NTA or anti‐GFP affinity beads (Smart‐Lifesciences). The eluted protein fractions were then concentrated, and the buffer was exchanged utilizing Amicon Ultra centrifugal filters (Millipore). The quality of the purified proteins was verified utilizing SDS‐PAGE (Figure S2), and the proteins were preserved in the aforementioned lysis buffer containing 10% glycerol at −80°C.

### 2.5 In Vitro Phase Separation Assay

Purified EGFP‐tagged fusion proteins were prepared in a buffer solution enriched with 50 mM Tris‐HCl (pH 7.5) with varying salt concentrations. To examine the impact of EGR1's target DNA on its phase separation, 100 bp double‐stranded DNA fragments from the CDK4 promoter region, labeled with TAMRA (Ruibio), were incorporated. The protein‐DNA mixtures were then placed into a 384‐well microscopy plate (Cellvis), followed by sealing employing an optically clear adhesive film (Cellvis). Observations were made utilizing a Nikon AX R Confocal Microscope System using a 63× oil—immersion objective with a numerical aperture (NA) of 1.4. For fluorescence imaging, a 488 nm laser line was used for excitation at 30% laser power, and the emitted light was detected in an appropriate wavelength range depending on the specific fluorophores being studied. The acquisition was performed using the Nikon AX R system's dedicated software, and the images were subsequently processed with Fiji software.

### 2.6 FRAP Analysis

This analysis was made with the Nikon AX R Confocal Microscope System with a 100× oil immersion objective. A specific section of the protein droplets was subjected to bleaching for ≈2–3 s using a 488 nm laser set at 80% intensity. Post‐bleaching, the recovery of fluorescence intensity was monitored and recorded at the indicated time points. The fluorescence intensities of photobleached areas were normalized with the intensities of unbleached areas. The analysis of fluorescence recovery was implemented utilizing the Nikon AX R system's dedicated software, noting the recovery duration over a specified period.

### 2.7 Statistical Analysis

Data were processed employing GraphPad Prism software, with results summarized as mean ± SD. Statistical significance was determined utilizing Student's *t*‐test or one‐way ANOVA, followed by Tukey's multiple comparisons test. A *P*‐value of less than 0.05 was deemed significant for all tests.

## 3 Results

### 3.1 Highly Expressed EGR1 in ADPKD Kidneys

**FIGURE 1:**
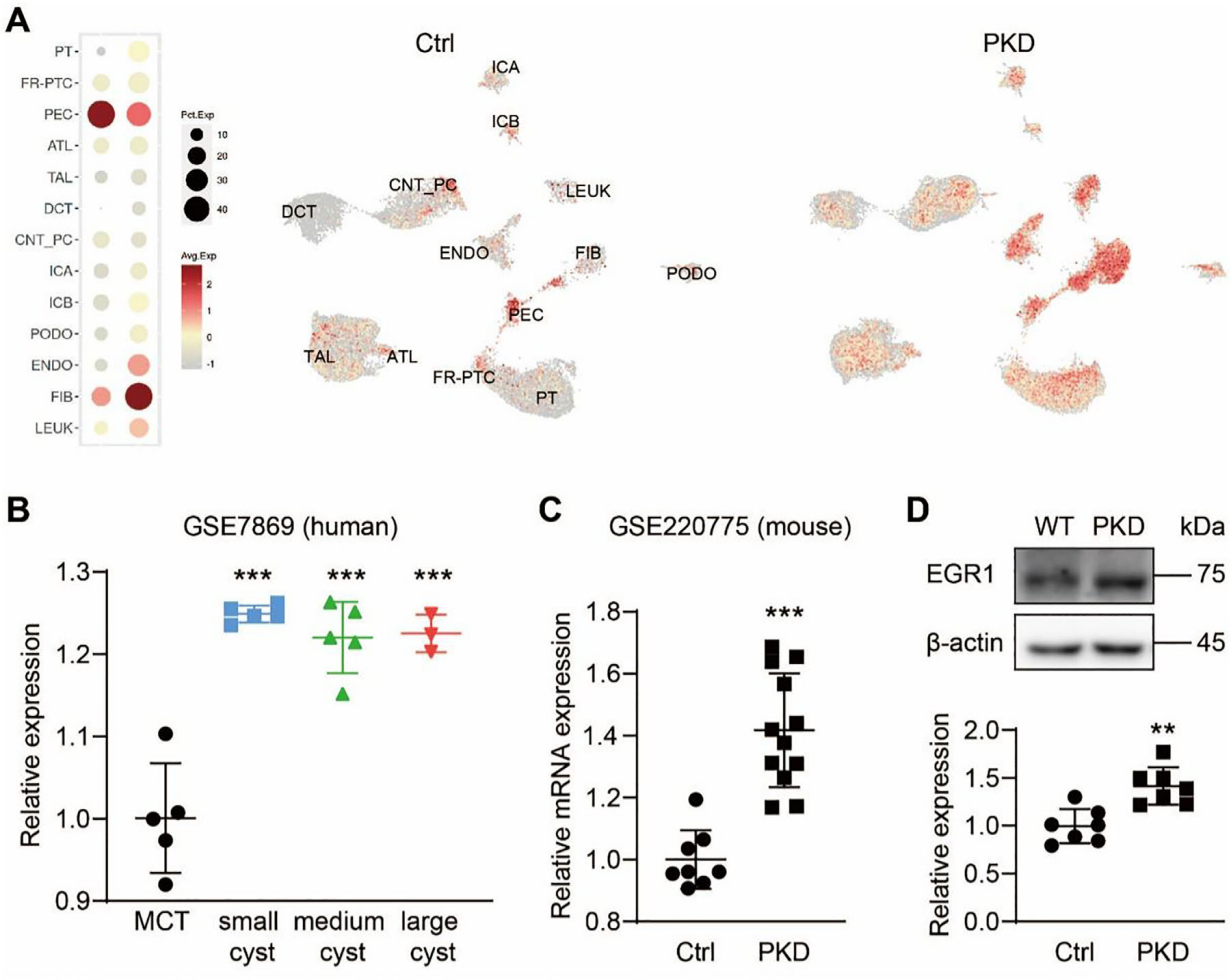
EGR1 expression in normal and ADPKD kidneys. (A) EGR1 expression levels in human normal and ADPKD renal cells. (B) Relative mRNA levels of EGR1 in human ADPKD cysts of different sizes compared to minimally cyst tissue. (C) Relative mRNA levels of *Egr1* in kPKD mouse kidney tissue. (D) Typical Western blots of EGR1 in wild‐type (WT) and kPKD kidney tissue. *n* = 6. All data are detailed as means ± SD. *P < 0.05, **P < 0.01, ***P < 0.001 versus Ctrl group.

To explore the levels and significance of EGR1 in ADPKD, we analyzed three gene expression datasets: GSE185948, GSE7869, and GSE220775. A detailed analysis of a single‐cell sequencing study (GSE185948) [35] revealed that *EGR1* was markedly upregulated in various cell types within the human ADPKD kidneys, notably in the proximal tubules (PT), parietal epithelial cells (PEC), thick ascending limbs of Henle, and distal convoluted tubules (Figure 1A). Additionally, we observed an enhanced expression of *EGR1* across renal cysts of varying sizes in ADPKD patients (Figure 1B). In parallel, data from a mouse mRNA sequencing series (GSE220775) indicated a notable rise in *Egr1* expression in the kidneys of mouse models of ADPKD (Figure 1C). Western blot analysis validated that EGR1 levels were 1.45‐fold higher in kidney‐specific *Pkd1*‐deficient mice compared to control mice (Figure 1D). These findings collectively underscore the consistent overexpression of EGR1 in both human and mouse models of ADPKD.

### 3.2 EGR1 inhibitor Impeded In Vitro Cyst Development

**FIGURE 2:**
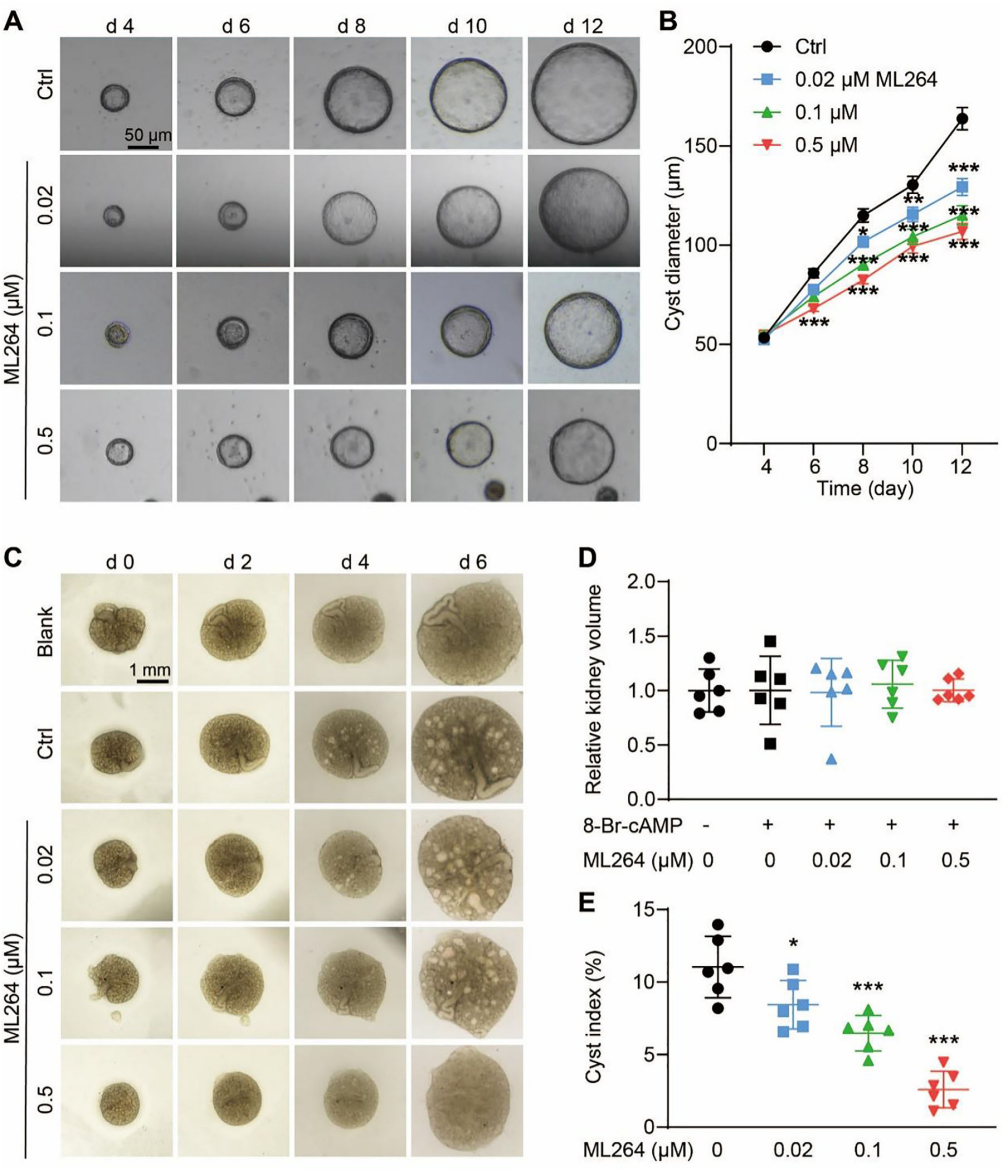
Effect of EGR1 inhibitor on in vitro cyst development. (A) Typical images of MDCK cysts in Matrigel without or with different concentrations (0.02, 0.1, and 0.5 µM) of ML264 treatment from day 4 to day 12. Bar = 50 µm. (B) Cyst diameters in conditions described in (a). *n* = 35. (C) Typical images of embryonic kidney cysts incubated with 8‐Br‐cAMP and without or with different concentrations (0.02, 0.1, and 0.5 µM) of ML264 from day 0 to 6. *n* = 6. (D) Relative kidney volume of embryonic kidneys treated without or with ML264 on day 6. (E) Measurement of the cyst index in embryonic kidneys with and without ML264 treatment on day 6. All data are detailed as mean values ± SD, *n* = 6. *P < 0.05, **P < 0.01, ***P < 0.001 versus Ctrl group.

Then, the MDCK cyst model stimulated with FSK was developed for exploring the potential of EGR1 in cystogenesis. Our observations revealed that the EGR1 inhibitor, ML264, markedly limited cyst growth in a concentration‐dependent fashion (Figure 2A,B). This suppression of cyst enlargement by ML264 was also confirmed using the embryonic kidney cyst model. Notably, cyst formation was evident in embryonic kidneys by day 4 post‐culture. Over a period from day 0 to day 6, while ML264 did not alter the overall growth of the embryonic kidneys (Figure 2C,D), it effectively decelerated the expansion of cysts, as demonstrated by changes in the cyst ratio (Figure 2C,E). These findings highlight the contributory role of EGR1 in promoting cyst development driven by cAMP, both in vitro and ex vivo.

### 3.3 EGR1 Inhibition Alleviated Renal Cyst Development In Vivo

**FIGURE 3:**
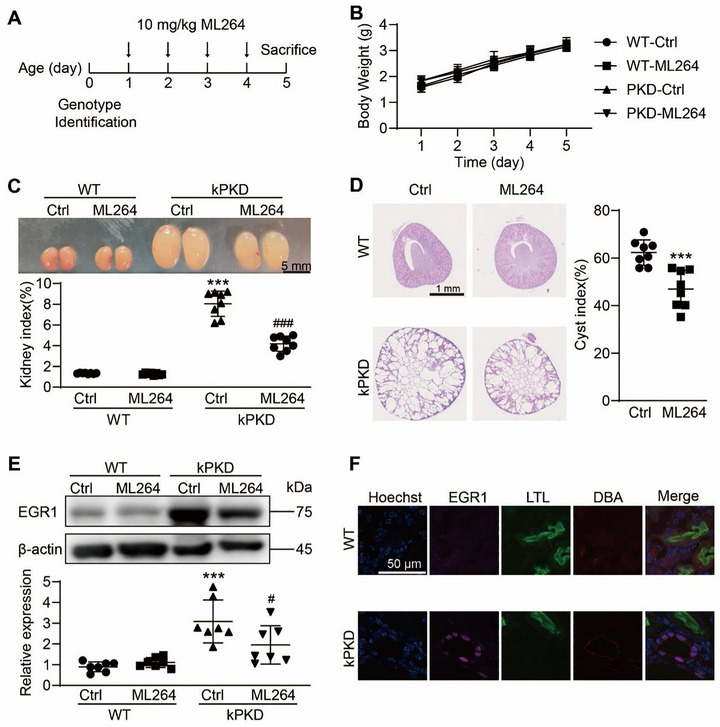
Effect of EGR1 inhibitor on mouse renal cyst development. (A) Genotyping and drug administration schematics. (B) Growth curves of mice. *n* = 7–8. (C) Typical photographs of wild‐type (WT) and kPKD kidneys treated without (Ctrl) or with ML264 at 10 mg kg^−1^ per day from postnatal day 1 to day 5. Bar = 1 cm. Also shown is the kidney index, which is the ratio of kidney weight to total body weight, for each group. *n* = 7–8. ***P < 0.001 versus WT Ctrl mice. ^###^ *P* < 0.001 versus kPKD Ctrl mice. (D) Typical kidney tissue sections following H&E staining from kPKD mice treated without (left) or with ML264 (right). Bar = 1 mm. The right panel quantifies the cyst index, defined as the percentage of cyst area relative to total kidney area. *n* = 7–8. ***P < 0.001 versus Ctrl group in kPKD mice. (E) Typical blots of EGR1 in the kidneys of WT and kPKD mice treated without or with ML264 (upper). Quantification of EGR1 expression (lower). *n* = 7–8. ***P < 0.01 versus WT Ctrl mice, ^#^ *P* < 0.05 versus kPKD Ctrl mice. (F) Immunofluorescence of EGR1 on kidney tissue sections from WT or kPKD mice. Nuclei were stained with Hoechst. Bar = 10 µm. All data are detailed as mean values ± SD.

Our next focus was the significance of EGR1 inhibition in renal cyst development. For this purpose, we utilized kidney‐specific PC1 knockout mice (kPKD) as our in vivo model (Figure 3A). Over a treatment period from postnatal day 1 to 4, administration of ML264 at a dosage of 10 mg/(kg day) did not notably impact the body weight of the mice (Figure 3B). In ML264‐treated kPKD mice, the kidney sizes and kidney indexes were significantly smaller than vehicle control kPKD mice (Figure 3C). Additionally, both the cyst sizes and the ratios of cystic to total kidney areas were substantially lower in the ML264‐treated group (Figure 3D). ML264 treatment also brought about a notable reduction in EGR1 expression in the kPKD kidneys compared to vehicle‐treated controls (Figure 3E). Immunofluorescence studies revealed a stark contrast in EGR1 localization, showing diffuse cytoplasmic staining in normal kidneys versus pronounced nuclear staining with distinct punctate formations in the cyst‐lining cells of kPKD mouse kidneys (Figure 3F), suggesting that EGR1 forms nuclear condensates in cystic kidneys. Collectively, these findings underpin the critical role of nuclear‐enriched EGR1 in driving ADPKD pathogenesis.

### 3.4 EGR1 Formed Nuclear Condensates in Human Cyst Epithelial Cells

**FIGURE 4:**
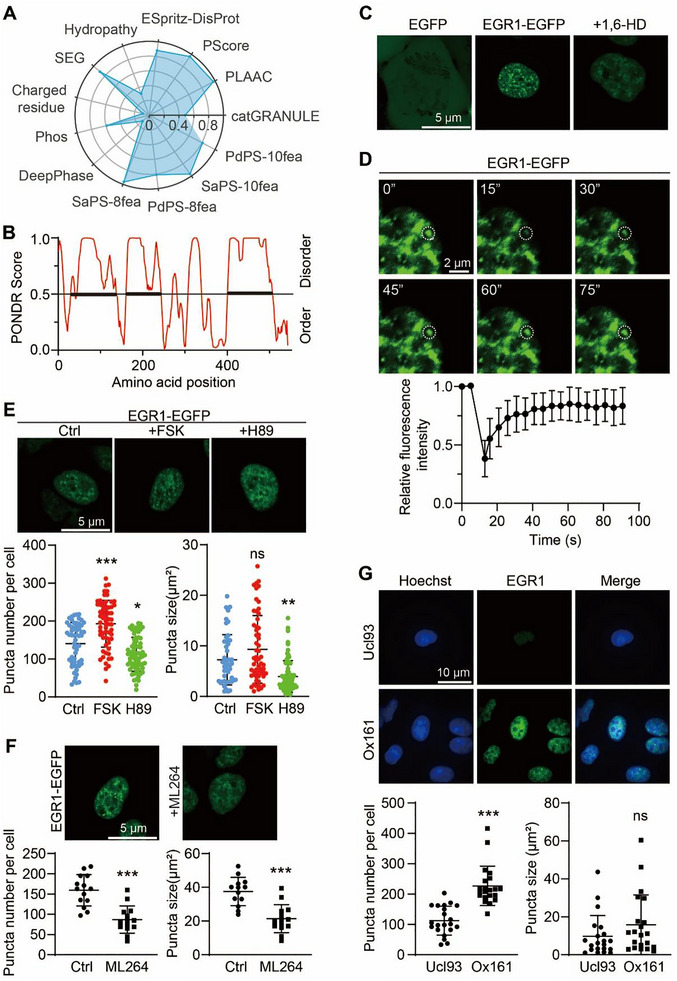
EGR1 nuclear condensates in human cyst epithelial cells. (A) A comprehensive profile of EGR1's phase separation predictors as ranked on the PhasePred platform (http://predict.phasep.pro/). Espritz‐disprot, accurate and fast prediction of protein disorder. *P*‐score, pi‐contact propensity; PLAAC, prion‐like composition; catGRANULE, granule‐forming propensity; SEG, ratio of local complexity in amino acid sequences and sequence databases. DeepPhase, proteome‐scale analysis of phase‐separated proteins in immunofluorescence images. Phos, the number of Phos sites. Charged residue, the fraction of the sequence of charged amino acids (D/E/R/K). SaPS, self‐assembling propensity. PdPS, partner‐dependent propensity. The 8fea model integrates eight sequence‐based features, including hydropathy, FCR, *P*‐score, PLAAC, espritz‐disprot, LCD, deepcoil, and catGRANULE. The 10fea model extends the 8fea by including Phos frequency and DeepPhase, two additional features. (B) The graph displays the intrinsic disorder of EGR1 based on the VL‐XT algorithm available at (http://pondr.com/). According to PONDR predictions, a score over 0.5 suggests significant disorder. The diagram highlights regions identified as intrinsically disordered. (C) EGFP and EGR1‐EGFP in live HEK293T cells in the presence or absence of 1.5% 1,6‐HD. (D) Sequential FRAP recovery images alongside a graph depicting the recovery curve for nuclear EGR1‐EGFP condensates. The photobleached area is marked with a dotted circle. Bar = 2 µm. Data are detailed as means ± SD (n = 10). (E) Typical images of live cells expressing EGR1‐EGFP without or with FSK or H89 treatment (upper). Bar = 5 µm. Quantification of puncta number and size per cell (lower). *P < 0.05, **P < 0.01, ***P < 0.001, ns means not significant versus Ctrl group. (F) Representative images of live cells expressing EGR1‐EGFP without or with ML264 treatment (upper). Bar = 5 µm. Quantification of puncta number and size per cell (lower). ***P < 0.001 versus Ctrl group. (G) Immunofluorescence of EGR1 (green) in fixed Ucl93 and Ox161 cells (upper). Nuclei were stained with Hoechst. Bar = 10 µm. Quantification of puncta number and size per cell (lower). ***P < 0.001, ns means not significant versus Ucl93 cells. All data are detailed as mean values ± SD.

Based on the immunofluorescence analysis of ADPKD kidney, we hypothesized that EGR1 formed phase‐separated condensates in renal cyst epithelial cells, thereby activating the transcription of its target genes. To assess the phase separation potential of EGR1, we used the Phasepred platform (http://predict.phasep.pro) and achieved a high PS‐self score of 0.869 (Figure 4A). Proteins that undergo phase separation often contain intrinsically disordered regions (IDRs) [36]. EGR1 was found to have three such predicted IDRs via sequence analysis (Figure 4B).

To verify the formation of EGR1 condensates in vivo, we overexpressed EGFP‐tagged EGR1 in HEK 293T cells. Observations via live‐cell fluorescence microscopy indicated discrete nuclear puncta of EGR1, in contrast to the uniform distribution seen with the EGFP control (Figure 4C). The disruption of these structures by 1,6‐hexanediol (1,6‐HD), which targets hydrophobic interactions within phase‐separated entities, further supported the liquid condensate nature of EGR1 (Figure 4C). Additionally, rapid fluorescence recovery post‐photobleaching (78% within 60 s) underscored the dynamic nature of EGR1 condensates (Figure 4D).

The influence of intracellular cAMP was apparent, as FSK stimulated nuclear condensate formation, while the PKA inhibitor H89 diminished the formation and size of these condensates (Figure 4E). Treatment with the EGR1 inhibitor ML264 similarly reduced the number and size of EGR1 puncta, suggesting its potential mechanism of inhibiting cyst growth through impacting EGR1 phase separation (Figure 4F).

Further investigations using an EGR1‐specific antibody in various cell lines, including Ucl93, Ox161, and WT 9–12 human ADPKD cells, revealed visible nuclear puncta, with a higher count in Ox161 cells. The application of 1,6‐HD markedly disrupted the formation of EGR1 puncta in WT 9–12 cells (Figure S1A). Subsequent immunofluorescence experiments demonstrated that cAMP promoted, while 1,6‐HD dispersed, EGR1 condensates in mIMCD3 cell nuclei (Figure S1B). These observations collectively affirm the generation of EGR1 condensates in the nuclei of renal cystic epithelial cells in ADPKD.

### 3.5 EGR1 Underwent Phase Separation In Vitro

**FIGURE 5:**
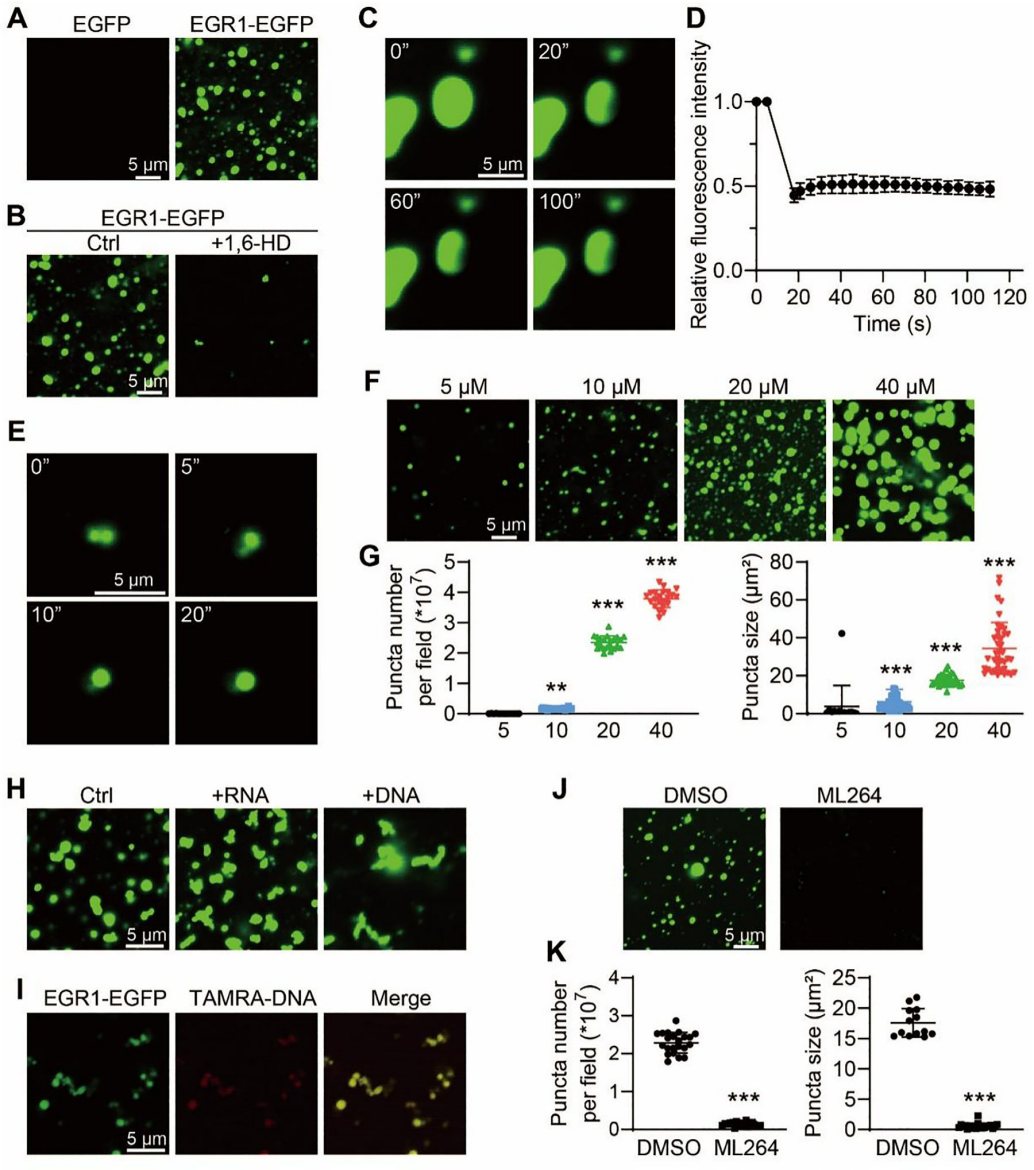
EGR1 phase separation in vitro. (A) Typical images illustrating droplet formation in EGFP and EGR1‐EGFP. (B) Typical images of depicting the formation of EGR1‐EGFP droplets both with and without the addition of 1.5% 1,6‐HD. (C) Typical images of the in vitro FRAP experiment with recombinant EGR1‐EGFP. (D) Graphical representation showing the dynamics of fluorescence recovery in EGR1‐EGFP droplets post‐photobleaching. (E) Time‐lapse micrographs showing the merging of EGR1‐EGFP droplets. (F) Representative images depicting droplet formation across various concentrations of EGR1‐EGFP. (G) Quantification of droplet number and size per field at specified EGR1‐EGFP concentrations. **P < 0.01, ***P < 0.001 versus 5 µM EGR1‐EGFP group. (H) Typical images of droplet formation of EGR1‐EGFP without or with RNA or DNA treatment. (I) Typical images of droplet formation of EGR1‐EGFP with TAMRA‐modified DNA. (J) Typical images showing the impact of ML264 treatment on EGR1‐EGFP droplet formation. (K) Quantification of droplet number and size per field of EGR1‐EGFP without or with ML264 treatment. Bar = 5 µm for all representative images. ***P < 0.001 versus Ctrl group. *All data are detailed as mean values ± SD*.

To test whether EGR1 forms phase‐separated droplets in vitro, we purified recombinant EGR1‐EGFP fusion protein expressed in E. coli (Figure S2B). Fluorescence microscopy demonstrated that these proteins formed micron‐sized, spherical droplets, in contrast to the uniformly dispersed EGFP alone (Figure 5A). Furthermore, such droplets are sensitive to the hydrophobic interference of 1,6‐HD, showing droplet dissipation (Figure 5B). Further analysis using fluorescence recovery after photobleaching on these droplets showed limited internal mobility within EGR1, suggesting a more solid‐like state rather than a fluid‐like state (Figure 5C,D). Droplet formation assays revealed that EGR1‐EGFP showed droplet fusion (Figure 5E) and formed phase‐separated droplets in a concentration‐dependent fashion, higher concentrations of EGR1 formed more and larger droplets (Figure 5F,G), while such droplets were dampened by a high NaCl concentration (Figure S2A). We then focused on determining whether the phase separation of EGR1 as a TF would be regulated by DNA and RNA. It was found that RNA and DNA served as a glue to hold EGR1 droplets together, and DNA fragments modified with TAMRA containing 5′GCGGGGGCG3′ sequences could be cohesively coalesced in EGR1 droplets, indicating EGR1 phase separation contributes to the assembly of transcriptional condensate (Figure 5H,I). Besides, EGR1 inhibitor ML264 limited the number and size of EGR1 droplets in vitro (Figure 5J,K), implying that ML264 may exert its inhibitory effect on EGR1 by affecting EGR1 condensate formation again.

### 3.6 Essential Role of the DNA Binding Domain (DBD) in EGR1 Phase Separation

**FIGURE 6:**
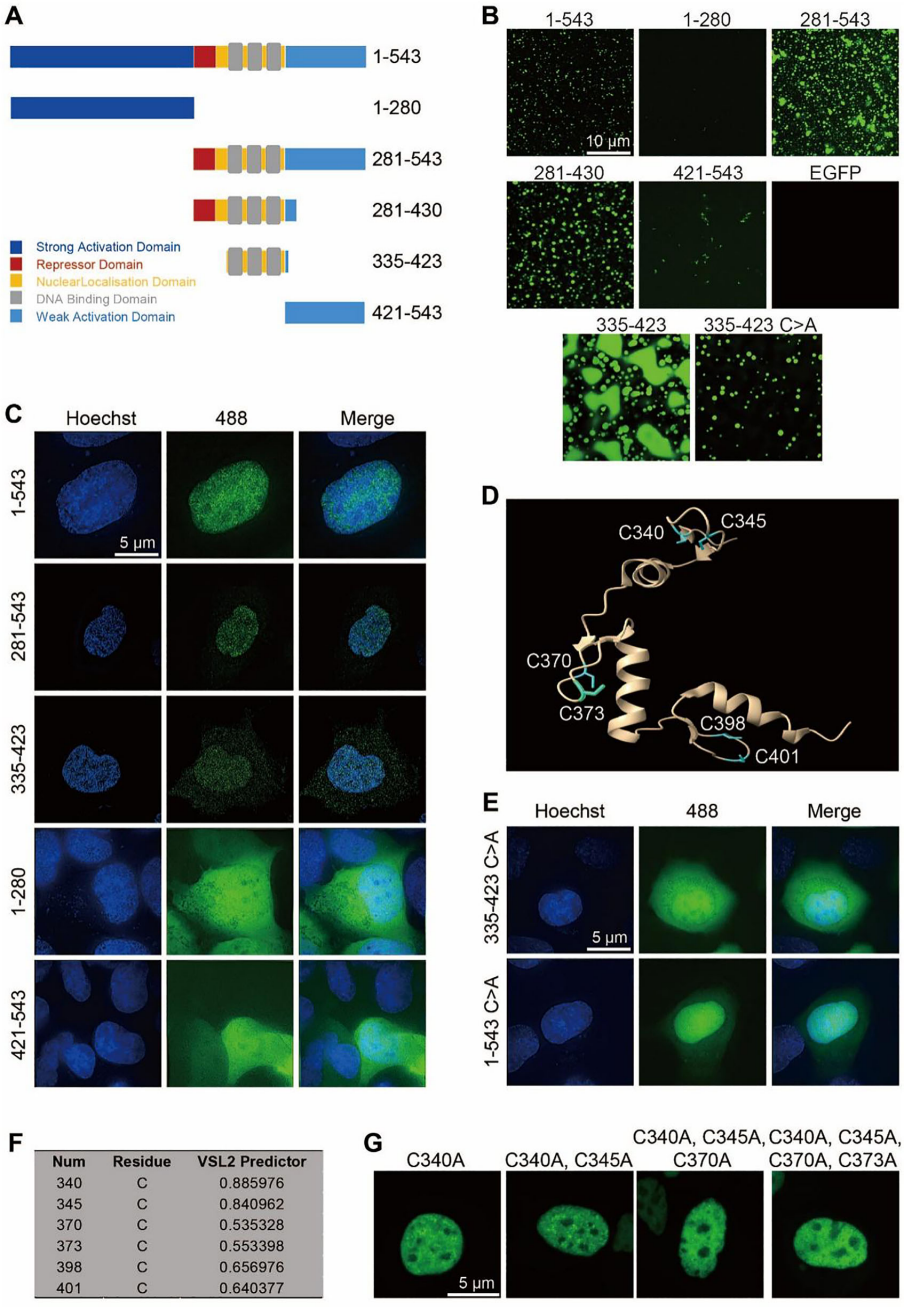
The effect of DNA‐binding domain in EGR1 phase separation. (A) Schematic diagram for EGR1‐EGFP truncations. (B) Representative images illustrating droplet formation of different truncations. Bar = 10 µm. (C) Typical images showing live‐cell expressing EGR1‐EGFP truncations. *Nuclei were stained with Hoechst*. Bar = 5 µm. (D) The positions of cysteine residues in the crystal structure of EGR1. (E) Representative images of EGR1 with the disruption of the zinc finger motif (C>A) fused to EGFP in live‐cell puncta formation assay. All cysteines in the zinc finger motif were mutated to alanines. *Nuclei were stained with Hoechst*. Bar = 5 µm. (F) The PONDR score of cysteines in EGR1. (G) Representative images of EGR1 with different numbers of cysteine mutations fused to EGFP in a live‐cell puncta formation assay. Bar = 5 µm.

Typically, TFs are structured with a DBD and an activation domain (AD), each playing critical roles in gene regulation [37]. Transcriptional activation can often be attributed to the ability of ADs, which contain IDRs featuring low‐complexity sequences, to undergo phase separation [38]. EGR1 is uniquely composed of two ADs located at its N‐terminal (NTD) and C‐terminal (CTD) domains, along with a regulatory domain (RD) and a distinct DNA‐binding domain. The DBD of EGR1 is particularly characterized by three Cys2‐His2 (C2H2) zinc finger motifs, which are crucial for recognizing and binding specific target gene sequences to modulate their transcriptional activity [39]. To identify the domains in EGR1 driving its phase separation, basing on the sequence analysis revealing that EGR1 has three predicted IDRs, we generated peptides 1–543 (the full length), 1–280 (the first and second predicted IDR), 281–543 (DBD and the third predicted IDR), 281–430 (RD and DBD), 335–423 (DBD), 421–543 (the third predicted IDR) of EGR1 truncations tagged with EGFP and purified recombinant proteins from a bacterial expression system (Figure 6A; Figure S2B). Of these peptides, only those containing DBD formed micron‐sized spherical droplets, mirroring the behavior of the full‐length protein, such as 281–543 (DBD and the 3rd predicted IDR), 281–430 (RD and DBD), and 335–423 (DBD) of EGR1‐EGFP (Figure 6B). To confirm the significance of DBD in condensate formation of EGR1 within cells, we tested their puncta formation capability by transfecting EGR1 truncations in the eukaryotic expression vectors into HEK293T cells. The cellular experiments confirmed the in vitro droplet formation results; truncations containing the DBD, unlike those with the IDR, NTD, or CTD, successfully formed nuclear puncta. Absence of the DBD in EGR1 constructs resulted in a failure to undergo phase separation (Figure 6C). These findings unveil the significance of the DBD in the phase separation of EGR1. The DBD consists of three C2H2‐type zinc fingers, each characterized by a pair of cysteine residues crucial for its structural configuration (Figure 6D).

The zinc finger motifs have been previously identified as facilitators of phase separation [40, 41]. To investigate this role further, we mutated the cysteines in the zinc finger motifs to alanines. Although this change markedly decreased EGR1's phase separation capacity, it did not completely prevent it in vitro or in cellular condensate formation (Figure 6B lower, E). This suggests that the structural integrity of the zinc finger motifs within the DBD is instrumental for its phase‐separating behavior. To further investigate whether different numbers of cysteine mutations would have an effect on EGR1 phase separation, according to the results of the VSL2 predictor (Figure 6F), we generated four EGR1 protein variants with mutations EGR1 C340A, EGR1 C340A‐C345A, EGR1 C340A‐C345A‐C370A, and EGR1 C340A‐C345A‐C370A‐C373A, and observed the puncta formation in cells. Live‐cell fluorescence microscopy revealed that mutating cysteine residues in EGR1 reduced its ability to undergo phase separation, with the impact being particularly pronounced when more than three specific sites in the EGR1 sequence were altered (Figure 6G). Together, these results strongly suggest that cysteine residues in DBD are required for EGR1 phase separation.

### 3.7 Phase Separation of EGR1 Promoted Hyperproliferation of Cystic Epithelial Cells by Facilitating the Transcription of Genes Related to Cell Cycle

**FIGURE 7:**
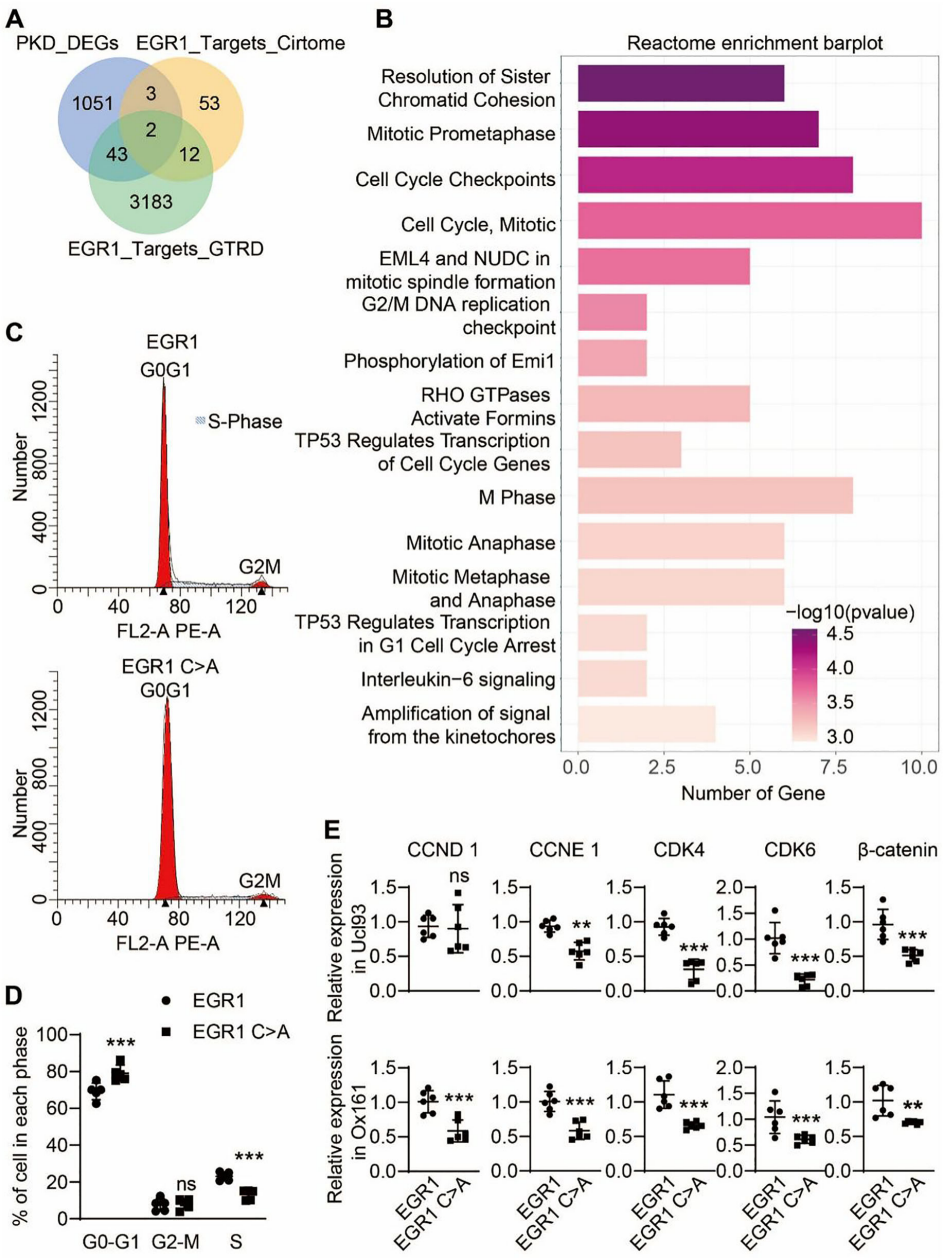
Biological functions of EGR1 phase separation. (A) Venn diagram depicting the intersection of differentially expressed genes (DEGs) in PKD with EGR1 target genes compiled from both Cistrome and GTRD databases. (B) Enrichment analysis of Reactome pathways for a set of 48 genes intersecting PKD DEGs and EGR1 targets. (C) The distribution of the cell cycle in Ox161 cells stably expressing EGR1 and EGR1 C>A tagged with EGFP was assessed by flow cytometry. (D) Quantifications of the proportion of cells in each stage. *n* = 6. ***P < 0.001, ns means not significant versus Ox161 cells stably expressing EGR1‐EGFP. (E) RT‐qPCR analysis of mRNAs for cell cycle‐related genes in Ucl93 and Ox161 cells stably expressing EGR1 or EGR1 C>A tagged with EGFP. *n*  =  6. **P < 0.01, ***P < 0.001, ns means not significant versus cells stably expressing EGR1‐EGFP. All data are detailed as mean values ± SD.

To explore the function of aberrant EGR1 condensate in ADPKD, we first used differentially expressed genes in ADPKD (GSE185948), the GTRD‐predicted EGR1 target genes, as well as the EGR1 ChIP‐seq database provided by Cistrome DB to search for the intersection of these data to identify which gene transcription related to polycystic kidney may be affected by EGR1. The results showed 48 intersecting genes (Figure 7A). Further analysis by Reactome enrichment demonstrated that these genes were clustered in cell cycle‐related pathways (Figure 7B). To further validate that EGR1 phase separation affects the cell cycle of cystic renal epithelial cells, we stably expressed EGR1 and EGR1 C>A mutants, defective in phase separation, in Ucl93 and Ox161 cells, and tested the potential of EGR1 phase separation on the cell cycle of Ucl93 and Ox161 cells. It was evident that EGR1 C>A mutants controlled the progression of Ox161 cells through the cycle by limiting S‐phase and reducing cells in the G0/G1‐phase (Figure 7C,D), whereas no significant difference was noted in the cell cycle of Ucl93 before and after EGR1 mutation (Figure S3C).

Next, we investigated the potentials of EGR1 condensates on the transcription of genes associated with the cell cycle, discovering that phase‐separated EGR1 significantly upregulated genes such as CCND1, CCNE1, CDK4, CDK6, and β‐catenin. This enhancement was particularly pronounced in Ox161 cells compared to Ucl93 cells (Figure 7E). To determine if the inability of the C‐A mutant to undergo phase separation underlies EGR1's reduced impact on cell proliferation, we employed the CCK‐8 assay. The results verified enhanced proliferation in Ox161 cells, which was significantly curtailed by the EGR1 mutant lacking phase separation capabilities; however, the normal proliferation rates in Ucl93 cells remained unaffected (Figure S3B). These observations suggest that EGR1 mutants deficient in phase separation can suppress the excessive proliferation of cystic epithelial cells by modulating cell cycle regulation.

### 3.8 EGR1 Formed Condensates With YAP1 and CBP/P300 to Promote Transcriptional Activation of CDK4

**FIGURE 8:**
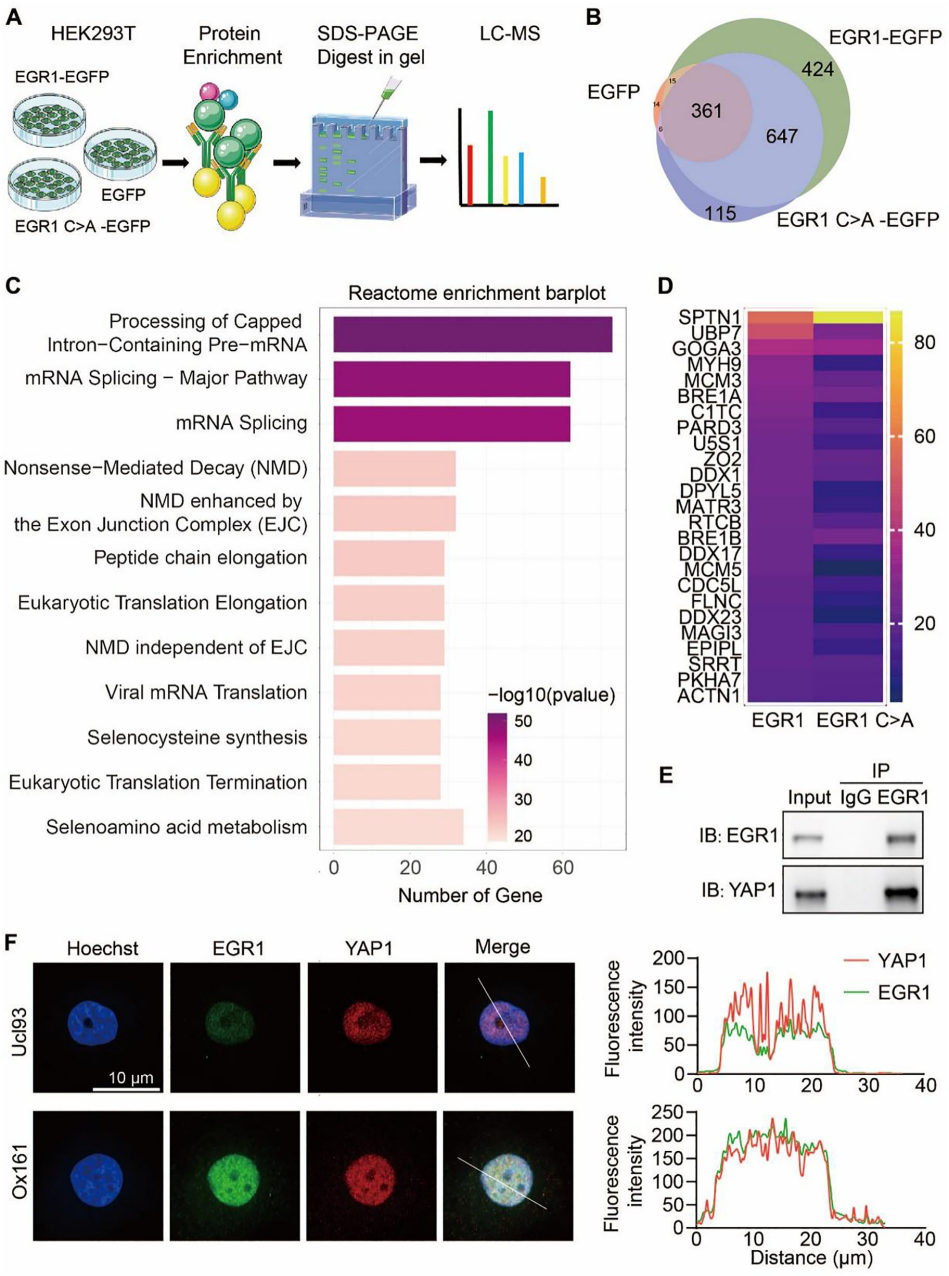
Condensate of EGR1 with YAP1. (A) Diagram illustrating the mass spectrometry approach used to identify proteins interacting with EGFP, EGR1‐EGFP, and EGR1 C>A‐EGFP. (B) Diagram showing both common and unique proteins interacting with EGR1‐EGFP and EGR1 C>A‐EGFP as revealed by mass spectrometry. (C) Enrichment analysis of Reactome pathways of 647 shared interaction partners of EGR1 and EGR1 C>A. (D) Heatmap comparison of peptide quantities identified in shared proteins between EGR1 and EGR1 C>A groups. (E) Co‐IP assay testing the interaction between EGR1 and YAP1. (F) Immunofluorescence of the co‐localization of EGR1 and YAP1 in Ucl93 and Ox161 cells (left). Line scans along the dotted lines in the images (right). Bar = 10 µm.

To elucidate the specific interactions that EGR1 engages in within cyst epithelial cells to drive their proliferation in ADPKD, we initiated a search for proteins that partner with EGR1 in these condensates. Utilizing co‐immunoprecipitation followed by mass spectrometry (Co‐IP/MS), we analyzed protein complexes formed in HEK293T cells that had been modified to express either EGR1 tagged with RGFP or its mutant form, EGR1 C>A‐EGFP (Figure 8A). This approach identified 1071 proteins that were associated with EGR1 and 762 proteins interacting with the phase‐separation deficient mutant EGR1 C>A (Figure 8B), in which 647 proteins could bind both EGR1 and EGR1 C>A. Based on Reactome enrichment analysis, the most common enriched proteins were involved in mRNA processing and transcription, consistent with the function of EGR1 in regulating gene transcription (Figure 8C). Interestingly, we found a lower cofactor peptide number in EGR1 C>A group than in the EGR1 group (Figure 8D), indicating that EGR1 phase separation contributed to the transcription complex assembly.

In the cell lines of Ucl93, OX161, and those stably expressing EGR1 and EGR1C>A, EGR1 siRNA significantly downregulated the mRNA expressions of both YAP1 and CBP. Subsequently, YAP1 siRNA decreased the mRNA expressions of EGR1 and CBP. Additionally, CBP siRNA downregulated the mRNA expressions of YAP1 and CBP as well (Figure S4A). Specifically, the knockdown of EGR1, YAP1, and CBP all lowered CDK4 mRNA levels (Figure S4A). ChIP‐qPCR was performed to examine the binding of EGR1 and EGR1 C>A to the promoter region of CDK4. The % input value for the binding of EGR1 to the promoter region of CDK4 was approximately 0.6, while the % input value significantly decreased to 0.35 in the EGR1 C>A group (Figure S4B). These data indicate that the EGR1 C‐A mutant inhibits the binding of EGR1 to the promoter of CDK4, potentially reducing the transcriptional activation of CDK4.

Co‐immunoprecipitation analysis showed that endogenous EGR1 interacted with YAP1 in HEK 293T cells (Figure 8E) and EGR1 interacted with YAP1 or CBP in Ox161 cells (Figure S4C). Similarly, when elucidating the endogenous proteins in Ucl93 and Ox161 cells, it was evident that most EGR1 puncta stained by its antibody overlapped with YAP1 signal, and the co‐localization of EGR1 condensates with YAP1 was more visible in the nucleus of Ox161 cells (Figure 8F). Besides, in Ox161 cells stably expressing EGR1 or EGR1 C>A tagged with EGFP, most EGR1 puncta overlapped with YAP1 or CBP, while EGR1 C>A was widely and evenly distributed in the nucleus, and the co‐localization of EGR1 condensates with YAP1 or CBP was more visible in the EGR1 group (Figure S4D,E).

**FIGURE 9:**
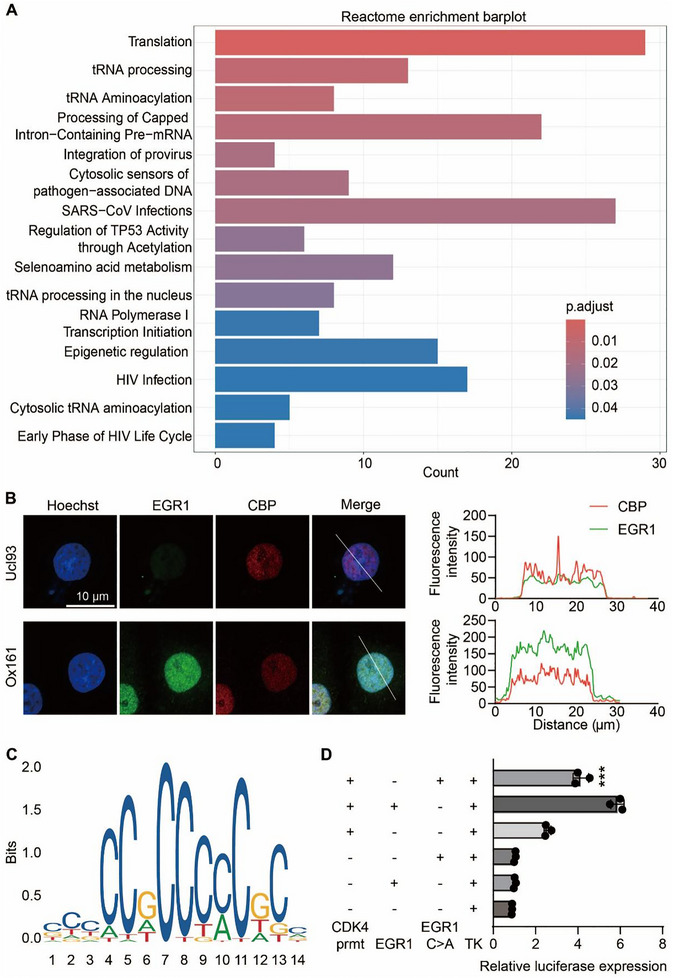
Condensate of EGR1 with CBP. (A) Enrichment analysis of Reactome pathways of specific protein interaction partners of EGR1. (B) Immunofluorescence of the co‐localization of EGR1 and CBP in Ucl93 and Ox161 cells (left). Line scans along the dotted lines in the images (right). (C) The sequences of EGR1 transcriptional binding sites and the predicted binding sequences on the CDK4 promoter region. (D) Relative luciferase activity in different groups was examined by a luciferase assay. *n* = 3. All data are detailed as mean values ± SD. ***P < 0.001 versus EGR1 group.

Further analysis revealed that the disruption of EGR1's phase separation capacity resulted in the loss of interactions with proteins involved in translation, tRNA processing, and tRNA aminoacylation (Figure 9A). Among the identified interaction partners, we specifically emphasized the significance of the CBP, which is crucial in histone acetylation. Immunofluorescence analysis revealed that CBP co‐localized with EGR1 in Ucl93 and Ox161 cells (Figure 9B), demonstrating that interactions between CBP and EGR1 may be phase‐separation‐dependent. To evaluate the targeting effectiveness of EGR1 condensates, a luciferase assay was utilized. The CDK4 promoter, harboring several potential EGR1 binding sites as identified by the Jaspar database (Jaspar.genereg.net), was integrated upstream of the transcription start site into the pGL3 plasmid, resulting in the construction of the pGL3‐CDK4 reporter plasmid (Figure 9C).

The luciferase assay revealed that phase‐separated EGR1 markedly increased luciferase activity, indicating successful targeting of the CDK4 promoter region (Figure 9D). Collectively, these results demonstrate that EGR1 condensates, incorporating coactivators such as YAP1 and CBP, significantly boost EGR1's transcriptional activity by binding to and regulating the expression of downstream target gene promoters.

### 3.9 Disruption of EGR1 Phase Separation Significantly Alleviated Cyst Growth

**FIGURE 10:**
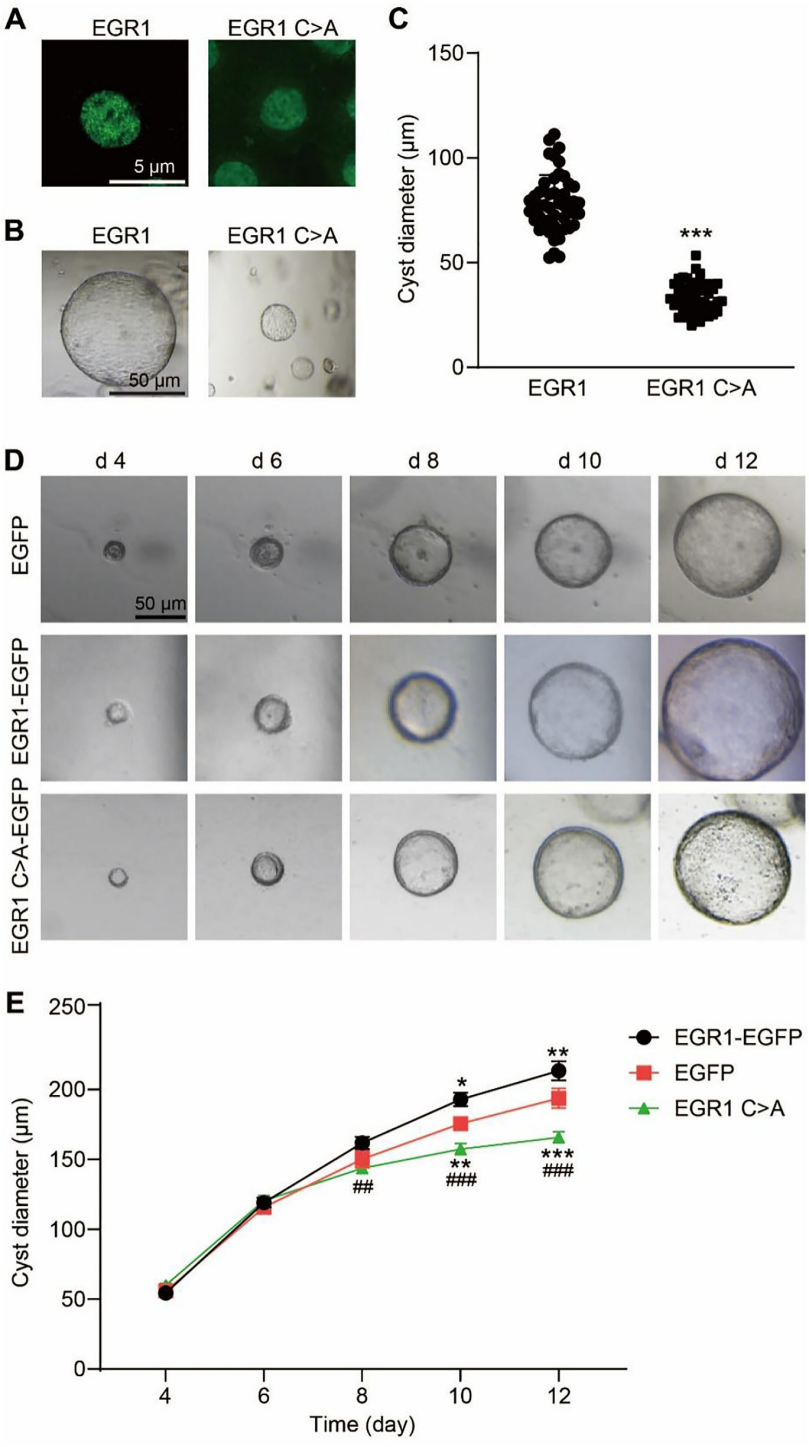
Effect of EGR1 phase separation on cyst development. (A) Representative immunofluorescence images of mIMCD3 cells expressing EGR1 or EGR1 C>A. (B) Representative images of a 3D spheroid of mIMCD3 cells expressing EGR1 or EGR1 C>A on day 3 of culture. Bar = 50 µm. (C) Diameters of cysts formed by mIMCD3 cells expressing EGR1 or EGR1 C>A. *n* = 35. ***P < 0.001 versus EGR1 group. (D) Typical images depicting cysts formed by MDCK cells stably expressing EGFP, EGR1‐EGFP, or EGR1 C>A‐EGFP. Bar = 50 µm. (E) Diameters of cysts formed by MDCK cells stably expressing EGFP, EGR1‐EGFP, or EGR1 C>A‐EGFP. *n* = 35. *P < 0.05, **P < 0.01, and ***P < 0.001 versus EGR1 group. ##*P* < 0.01, ###*P* < 0.001 versus EGR1 C>A group. All d*ata are detailed as mean values ± SD*.

To validate whether the absence of phase‐separation capability of EGR1 could decelerate cyst development, we adopted a 3D spheroid model with mIMCD3 cells to develop epithelial spheroids. We seeded mIMCD3 cells transfected with EGR1 or EGR1 C>A in Matrigel to observe the relationship between nuclear puncta and FSK induced spheroid formation. Immunofluorescence analysis showed EGR1 C>A of the DBD countermanded the capability to create nuclear puncta in contrast to EGR1 puncta in mIMCD3 cells (Figure 10A). The deficiency of EGR1 phase‐separated ability retarded FSK induced cyst enlargement by approximately 50% (Figure 10B,C). To elucidate the longer‐term effect of EGR1 phase separation on cyst development, we cultured MDCK cells stably expressing EGR1 or EGR1 C>A tagged with EGFP (Figure S3D) with FSK stimulation for 12 days. It was found that EGR1 significantly promoted cyst growth compared with the control group, and its cyst growth‐promoting effect was notably suppressed when its phase‐separated ability was disrupted (Figures 10D,E). These experimental results confirm that the phase separation characteristic of EGR1 is critical in its cell proliferative activity and cyst development.

## 4 Discussion

**FIGURE 11:**
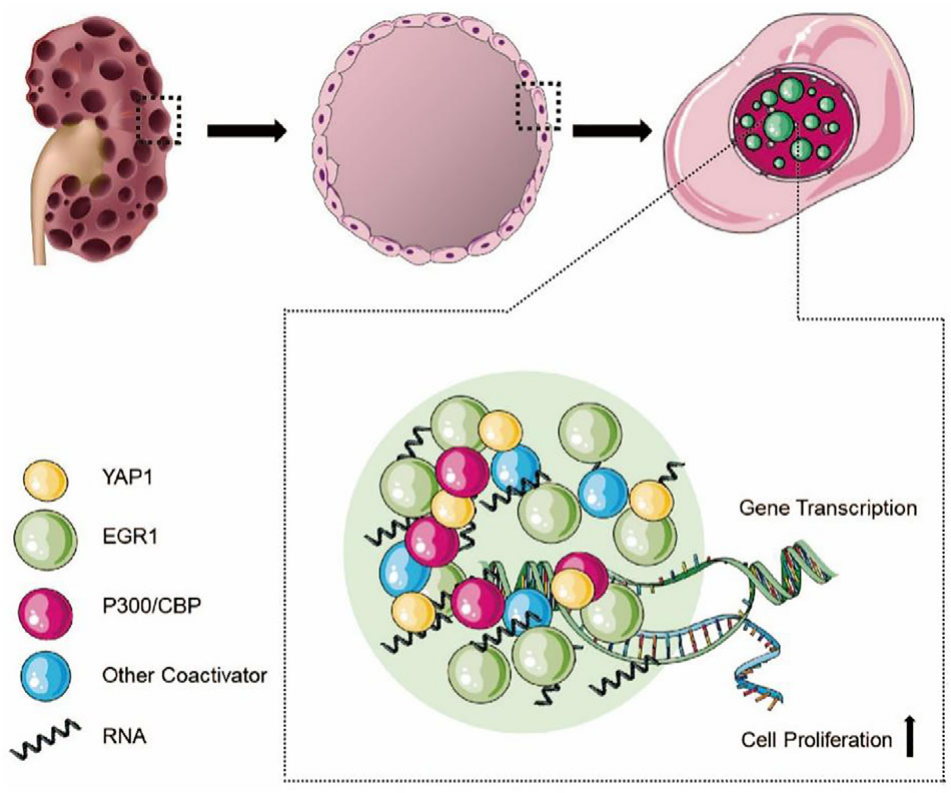
The proposed model highlights the crucial function of EGR1 condensates in the recruitment of co‐activators and facilitating promoter‐binding of EGR1, thereby initiating the expression of cell proliferation‐related genes to promote cyst development in PKD.

This research probed into the significance and underlying mechanisms of EGR1 in the development of renal cysts in ADPKD. Through various experimental approaches, including in vitro, ex vivo, and in vivo models specific to ADPKD, our findings underscore the significant impact of abnormal EGR1 phase separation in response to the cAMP‐PKA pathway on the progression of renal cysts in ADPKD (Figure 11).

Notably, renal cystic epithelial cells in ADPKD share pathophysiological traits with tumor cells, including abnormal proliferation and immature differentiation responding to the cAMP‐PKA pathway and the mitogenic signaling pathway [42]. Tumor cell proliferation hinges on cell cycle progression, relying on the interaction of cell cycle proteins with cell cycle protein‐dependent protein kinase (CDK), leading to the overactivation of D1‐Cdk4 and E‐Cdk2 [43]. Considering the vital role of cell proliferation in both tumorigenesis and cystogenesis, the search for key targets that regulate these proliferative signaling pathways is critical for the treatment of ADPKD. As a pivotal downstream factor of the cAMP‐PKA pathway, EGR1 possesses significant potential in regulating the proliferation of various tumor cell types, with a high expression in breast cancer, prostate cancer, and nephroblastoma [29, 44, 45, 46]. EGR1 has been documented to directly bind to the promoter region of cyclin D1 (CCND1), CCND2, max dimerization protein (MAD), and cyclin‐dependent kinase inhibitor 2D (CDKN2D), activating their transcription, positively regulating Pol I‐directed gene transcription, thereby expediting the cell cycle [16, 25, 47]. Consistently, we found high expression of EGR1 in human and mouse kidneys of ADPKD tissues and aberrant cell cycle‐related gene expression in ADPKD RNA‐seq. Moreover, EGR1 significantly promoted cyst growth in the MDCK cyst model, indicating the potential significance of EGR1 in the development of ADPKD.

Previous research has postulated that EGR1 orchestrates gene expression by directly binding to GC‐rich sequences in the promoter regions of its target genes [48]. However, given its expansive influence on downstream gene expression, the heightened levels of EGR1 alone may not entirely elucidate its diverse regulatory functions [49]. Moreover, the precise mechanisms through which EGR1 modulates cyst development in ADPKD remain elusive. Based on the aberrant enrichment of EGR1 in the nucleus of renal cyst epithelial cells of ADPKD mouse model, we hypothesize that EGR1 may exert its transcriptional regulation in a phase‐separated manner. Phase separation is critical in both signal transduction and the modulation of gene expression, especially in transcriptional regulation [50, 51, 52]. Liquid–liquid phase separation within different subcellular regions leads to the formation of membraneless condensates containing biomolecules, including proteins, RNA, and DNA, particularly the nuclear condensates that restrict multiple coactivators and act as super‐enhancers [50, 51, 53, 54, 55]. These condensates exhibit relevant biological activities, facilitating the processing of specific biological events in a relatively undisturbed manner [56, 57, 58]. For example, phase separation of Nrf2 is implicated in the modulation of redox balance in renal cyst cells [59]. By in vivo and in vitro phase separation assays, it was evident that EGR1 could undergo phase separation and form more condensates in the nucleus of renal cyst epithelial cells. Besides, EGR1 phase separation promoted the transcriptional activation and expression of target genes associated with the cell cycle, such as CCND1, CCNE1, CDK4, CDK6, and β‐catenin, promoting cell proliferation by inducing more cyst epithelial cells to enter the S phase and decreasing those in the G0/G1 phase. Our results offer a new insight into the transcriptional process regulated by EGR1, and partly explain the mechanism of EGR1 acting as an efficient TF to modulate the expression of numerous genes, especially during ADPKD development.

In fact, various TFs, including p53, GCN4, MYC, SOX2, NANOG, RARα, ERα, and GATA2, interact with mediator via the phase‐separating ability of their ADs, forming condensates with mediator engaged in gene activation [60, 61, 62]. In terms of specific cohesion components of EGR1 condensate, we focused on scrutinizing the interactions involving YAP1 and CBP with EGR1. YAP serves as a transcriptional co‐activator, critically influencing cell survival by engaging with specific enhancers to activate a set of target genes [60, 63, 64, 65, 66]. Recent studies have demonstrated that YAP can generate liquid‐like condensates in the nucleus. These condensates encapsulate TEAD1 and other associated co‐activators like TAZ, facilitating the activation of genes that promote cellular proliferation [67, 68]. It has been shown that EGR1 can interact with the WW structural domain of YAP1 through the PPxY motif within the 141–278 sequence region, and this interaction facilitates the transcriptional activity of EGR1 at the Bax promoter and mediates apoptosis [69]. Our study also showed that the cohesion formation of EGR1 and YAP1 is phase‐separated, interacting both before and after the EGR1 mutation, and further confirming that EGR1 does not interact with YAP1 through its DNA‐binding domain. While the coactivator p300/ CBP, whose promoter was transactivated by EGR1, facilitates gene regulation by promoting the assembly of transcriptional machinery and acetylating histones and other factors [44, 45]. It has been shown that NTD and CTD of CBP engage with the AD of EGR1 [70]. Our study validated the colocalization of EGR1 and CBP in renal epithelial cells, especially cyst epithelial cells, and mass spectrometry results showed that the co‐cohesion of CBP and EGR1 was phase‐separation‐dependent, and its co‐condensation was affected by the phase‐separation capacity of EGR1. However, the key region mediating the phase‐separation of EGR1 was not the transcriptional AD on its 1–280 sequence. Considering that CBP/p300 serves as a transcriptional activator when binding to DNA through the heterologous DNA‐binding domain and that the interaction between p300/CBP and EGR1 is a complex feedback loop. It is hypothesized that defective phase separation ability of EGR1 may affect the activation of CBP leading to an impact on the interactions between the two, although the exact mechanism needs to be further confirmed. Whatever, these findings further confirm the role of EGR1 condensate containing coactivators such as YAP1 or CBP in transcriptional regulation.

In this research, EGR1 variants with a disrupted zinc finger motif within the DBD demonstrated markedly reduced phase separation capabilities both in vitro and within cellular environments. The DBD in TFs typically acts as an anchor, selectively interacting with specific super‐enhancers or promoters to either initiate or boost transcription [37]. Meanwhile, ADs, which are rich in IDRs, aid in the assembly of transcriptional proteins via IDR‐driven phase separation, thereby influencing gene activation processes [38, 71]. Our findings indicated that the DBD, particularly through its zinc finger motif, was pivotal in promoting the phase separation of EGR1, more so than the IDR or AD components. Recent studies have underscored the zinc finger motif's similarity to IDRs in its ability to enhance protein oligomerization and facilitate phase separation [40, 41, 72]. Additionally, the unique arrangement of charged residues within EGR1's DBD, characterized by zinc finger structures, significantly contributes to its self‐assembly and phase separation propensity. This specific feature of the DBD, distinguished by zinc finger motifs and charged residue distribution, is crucial for EGR1's phase separation and may also influence other EGR isoforms like EGR2, EGR3, and EGR4, which share similar sequences in the DBD [73]. Moreover, the phase separation process of EGR1 is supported by the formation of disulfide bonds from cysteine, aligning with cystine's low solubility and the tendency of cystine‐rich peptides to produce structured aggregates via β‐sheet hydrogen bonding [74].

Furthermore, ML264, an inhibitor of EGR1, retarded cyst enlargement in our established three models, as well as impeded EGR1 phase separation both intracellularly and in vitro. ML264 is a small molecule compound that potently inhibits the expression of EGR1 and KLF5 in other contexts [75]. However, ML264 may exhibit off‐target effects. It could potentially interact with other transcription factors or proteins that share structural similarities with EGR1, or it may affect cellular processes that are not directly related to EGR1 function, such as cell cycle regulation or apoptosis. Moreover, considering that EGR1 is a member of the immediate early gene family [76], its dynamic expression during ADPKD progression reflects additional complexity.

In this study, the ADPKD mouse model exhibited severe polycystic kidney phenotypes by the fifth day after birth, which does not fully recapitulate the human disease course. Human ADPKD typically manifests later in life, with a more gradual progression of symptoms over many years or even decades. This accelerated disease progression in the mouse model may not accurately reflect the complex, long‐term pathophysiological changes and compensatory mechanisms that occur in human patients. This aspect has not been fully explored and deserves to be examined in detail in further research.

This research established a new model for understanding how EGR1 activates transcription, particularly of genes that regulate the cell cycle in ADPKD. It positions EGR1 not only as a central player in the pathology of ADPKD but also as a potential therapeutic target. The possibility of developing small‐molecule inhibitors that target EGR1 condensates offers a novel therapeutic strategy to curbing the excessive proliferation of cyst epithelial cells in ADPKD patients.

## Ethics approval statement

Animal experiments were conducted at Peking University Health Science Center. All experiments were performed in accordance with animal protocols approved by the Peking University Health Science Center Committee on Animal Research. Additionally, the Institutional Animal Care and Use Committee of Peking University Health Science Center provided approval for the animal experiments (Approval Number DLASBD0538).

## Conflicts of Interest

The authors declare that there are no conflicts of interest related to this study.

## Supporting information




**Supplementary File 1**: exp270107‐sup‐0001‐SuppMat.doc.

## Data Availability

Publicly available datasets analyzed in this study were obtained from NCBI GEO under accession numbers GSE185948 (https://www.ncbi.nlm.nih.gov/geo/query/acc.cgi?acc=GSE185948), GSE7869 (https://www.ncbi.nlm.nih.gov/geo/query/acc.cgi?acc=GSE7869), and GSE220775 (https://www.ncbi.nlm.nih.gov/geo/query/acc.cgi?acc=GSE220775). EGR1 ChIP‐seq data were retrieved from Cistrome DB (https://cistrome.org/db/), EGR1 target genes were compiled from GTRD (https://gtrd.biouml.org/), and motif information was obtained from JASPAR (https://jaspar.elixir.no/). Pathway and GO enrichment analyses used Reactome (https://reactome.org/) and the Gene Ontology Resource (https://www.geneontology.org/). Further data and additional details are available from the corresponding author upon reasonable request.
