# Supplementary material for: EGR1 Nuclear Condensates Promote Renal Cyst Development in Polycystic Kidney Disease
Source: Exploration (Beijing). 2025 Dec 26;6(1):20240285. doi: 10.1002/EXP.20240285 (PMC12970274; doi:10.1002/EXP.20240285)
Supplement: Supplementary file 1 — exp270107‐sup‐0001‐SuppMat.doc. [file EXP2-6-20240285-s001.doc]

Supplementary Methods

Supplementary Table S1

Supplementary Figures S1-S4

**Supplementary Methods**

***Gene Expression Omnibus (GEO) database extraction***

The GEO database (https://www.ncbi.nlm.nih.gov/geo/) is a public functional genomics data repository that deposits array- and sequence-based data. The keywords “ADPKD, human” or “ADPKD, mouse” were used to search the database and download related gene expression profiles. The mouse mRNA sequencing series dataset GSE220775 was analyzed using the DESeq2 package to identify differentially expressed genes (DEGs). The criteria for DEGs were set with a log fold change (logFC) of 2, indicating a 4-fold change in expression, and a p-value of 0.05 for statistical significance.

***Cell culture and CCK8 assay***

HEK293T cells and Madin-Darby Canine Kidney (MDCK) cells were cultured with Dulbecco’s modified Eagle’s medium (DMEM, Gibco) with 10 % fetal bovine serum (FBS), 100 μg/ml streptomycin, and 100 U/ml penicillin, incubated at 37 ℃ with 5% CO_2_. Mouse inner medullary collecting duct (mIMCD3) cells and WT 9-12 cells were cultured with Dulbecco's Modified Eagle Medium/Nutrient Mixture F12 (DMEM/F12, Gibco) with 10% FBS, 100 μg/ml streptomycin, and 100 U/ml penicillin, incubated at 37 ℃ with 5% CO_2_.. Human immortalized renal epithelial cells (Ucl93) and human-derived cystic renal epithelial cells (Ox161), purchased from BeNa Culture Collection and generated originally by A. C. Ong, University of Sheffield. (Sheffield, United Kingdom), were cultured in DMEM/F-12 (Gibco) with 10 %FBS, 100 μg/ml streptomycin, and 100 U/ml penicillin, incubated at 37 ℃ with 5% CO_2_.

To detect growth curves for different groups, cells were plated in 96-well plates at a density of 800 cells per well and incubated with CCK8 solution (Dojindo Molecular Technologies) at a 1:10 dilution at different time points for 30 minutes. Absorbance at 450 nm was measured with a microplate reader (MQX200; Biotek).

***Histological staining and immunofluorescence***

For hematoxylin and eosin (H & E) staining, 5 μm of kidney paraffin sections were used. Cyst diameter and cystic index were calculated using Image J NIH software (NIH Image, Bethesda, MD). Cystic area was measured based on the definition that a cyst was spherical structure with diameter ≥ 50 μm (cyst index = total cystic area/total kidney area) *100 %.

For immunofluorescence, sections were blocked with 5% (w/v) goat serum at room temperature for 1 h and then incubated with primary antibodies of EGR1(CST, #4153, rabbit, 1:1000), YAP1(abcam, ab52771, rabbit ,1:200) or CBP(CST, #7389, rabbit, 1:100) at 4 ℃ for overnight. After washing 3 times, sections were incubated with fluorophore-conjugated secondary antibodies (Proteintech) for 1 h. Hoechst dye 33342 (Invitrogen) was used to stain nuclei. All images were captured with the Nikon AX R Confocal Microscope System.

***3D spheroid model of mIMCD3 cells***

mIMCD3 cells were cultured in Matrigel with DMEM/F12 medium in the presence of or 10 μM FSK to induce cyst formation. The medium was changed every 12 h. Images were collected on d 3 using the Nikon Ti2-U. Cyst diameter was measured and analyzed Image J NIH software (NIH Image, Bethesda, MD).

***Live Cell Imaging***

HEK293T cells were seeded on glass-bottom cell culture dishes (Cellvis) and transfected with indicated plasmids. At 24th h after transfection, the cells were treated with FSK, ML264 or H89 for 1 h or not. Fluorescence images were captured on STELLARIS 8 FALCON FLIM microscope from Leica microsystems and analyzed with Image J NIH software (NIH Image, Bethesda, MD) to identify fusion events. The number and area of droplets or puncta were analyzed by aivia from Leica.

***Plasmid construction***

cDNA fragments encoding the proteins of interest were synthesized by Beijing Tsingke Biotech Co., Ltd. and amplified by PCR with PrimeSTAR Max DNA Polymerase (Takara), while cDNA coding EGR1 truncations (residues 1-280, residues 281-430, residues 281-543, residues 335-423, residues 335-423 C>A and residues 421-543) were generated by PCR from a plasmid containing full-length EGR1 with appropriate sets of primers. EZ-HiFi Seamless Cloning Kit (GenStar) was used to insert these sequences into the pET-22b and pcDNA3.1(+) vector containing EGFP tag. Plasmid inserts were confirmed by Sanger sequencing in Beijing Ruibio BiotechCo., Ltd, reading from both ends of the insert.

***Lentivirus production and transduction***

Lentiviral transfer constructs encoding EGR1-EGFP and EGR1 C>A-EGFP fragments were transfected with packaging plasmids into HEK293T cells using ExFect Transfection Reagent (Vazyme) according to the manufacturer’s instructions. Lentiviral supernatants were collected at 48^th^ h or 72^nd^ h after transfection. The pellets were dissolved with DMEM and stored at -80 °C. Ucl93 or Ox161 cells were infected by adding filtered viral supernatant. Cells stably expressing EGR1-EGFP or EGR1 C>A-EGFP were screened via FACS according to the fluorescence tag.

***Flow cytometry analysis of cell cycle***

For cell cycle experiments, cells were seeded in 6-well plates and resuspended with 500 μL 70% ethanol overnight at 4 °C. The next day, the cells were washed twice with PBS, resuspended with 500 μL PI/RNase staining buffer (BD Biosciences), and stained for 10 min at room temperature in darkness. The samples were washed twice again. Subsequently, the cell cycle was detected by the flow cytometry and analyzed it using Modfit software.

Chromatin Immunoprecipitation followed by Quantitative PCR (ChIP-qPCR)

Cells were cross-linked with 1% formaldehyde for 10 min at room temperature, followed by quenching with 125 mM glycine for 5 min. Cells were then washed twice with ice-cold phosphate-buffered saline (PBS) and lysed in ChIP lysis buffer (50 mM HEPES-KOH pH 7.5, 140 mM NaCl, 1 mM EDTA, 1% Triton X-100, 0.1% sodium deoxycholate, and 0.1% SDS) supplemented with protease inhibitors. Chromatin was sheared using a sonicator (Bioruptor, Diagenode) to achieve DNA fragments ranging from 200~500 bp. The sheared chromatin was centrifuged at 12,000 × g for 10 min at 4°C, and the supernatant was collected.

For immunoprecipitation, 5 µg of EGR1 antibody (CST, #4153, rabbit) or control IgG was incubated with 50 µL of Protein A/G magnetic beads (Thermo Fisher Scientific) for 2 h at 4°C. The pre-cleared chromatin was then added to the antibody-bead complex and incubated overnight at 4°C with rotation. Beads were washed sequentially with low-salt buffer (20 mM Tris-HCl pH 8.0, 150 mM NaCl, 2 mM EDTA, 1% Triton X-100, and 0.1% SDS), high-salt buffer (20 mM Tris-HCl pH 8.0, 500 mM NaCl, 2 mM EDTA, 1% Triton X-100, and 0.1% SDS), LiCl buffer (10 mM Tris-HCl pH 8.0, 250 mM LiCl, 1 mM EDTA, 1% NP-40, and 1% sodium deoxycholate), and TE buffer (10 mM Tris-HCl pH 8.0, 1 mM EDTA). Immunoprecipitated DNA-protein complexes were eluted in elution buffer (1% SDS, 100 mM NaHCO3) and reverse cross-linked by incubation at 65°C overnight. DNA was purified using a PCR purification kit (ChIP-IT High Sensitivity, Active Motif) and quantified using a NanoDrop spectrophotometer (Thermo Fisher Scientific) for subsequent qPCR.

siRNA Transfection and RNA Extraction for qPCR Analysis

Cells were seeded in 6-well plates and cultured overnight in complete medium until 30-40% confluency was reached. For transfection, siRNA-lipid complexes were prepared by diluting 20 µM siRNA and Lipofectamine 2000 in DMEM Reduced Serum Medium. Specifically, 2.5 µL of siRNA (final concentration 50 nM) and 30 µL of Lipofectamine 2000 were mixed in 500 µL of DMEM and incubated at room temperature for 15 minutes. The complexes were then added dropwise to the cells, followed by gentle swirling to ensure even distribution. After 24 hours of incubation at 37°C with 5% CO₂, cells were washed twice with ice-cold PBS and lysed directly in the wells using TRIzol (1 mL per well) for total RNA extraction. The lysates were collected, incubated at room temperature for 5 minutes, and stored at -80°C until further processing.

***RT-qPCR***

Total RNA was isolated from kidney tissue or cells using TRIzol (Invitrogen, 15 596 018). RNA was reverse transcribed by the cDNA Synthesis Kit (Vazyme). Sequences of primers are provided in Supplementary Table 1.

***Co-IP assay***

Co-IP assay were performed as described previously^34^. Cells were lysed with NP-40 lysis buffer (200 mM NaCl, 0.5% NP-40, 50 mM Tris-HCl pH 7.5, containing protease inhibitor cocktail). Corresponding antibodies were added into sample lysates and incubated overnight at 4 °C. Proteins were immunoprecipitated by Protein A/G Magnetic Beads (Selleck) overnight at 4 °C. The beads were washed with NP-40 lysis buffer 3 times. For Co-IP assay, the beads were boiled for 10 min for Western blotting analysis.

***Western blot***

Tissue or cell samples were collected and lysed using a radioimmunoprecipitation assay (RIPA) buffer containing 4% protease and 1% phosphatase inhibitors to prevent protein degradation and dephosphorylation. The lysates were then centrifuged at 4°C to remove cellular debris, and the supernatant was collected. Protein concentrations were determined using a bicinchoninic acid (BCA) protein assay kit (Thermo Fisher Scientific) according to the manufacturer's instructions. Protein samples were mixed with loading buffer and heated at 95°C for 5 minutes. Proteins were separated on 10 % SDS-polyacrylamide gel though electrophoresis at a constant voltage of 80 V for 0.3 hours and 120 V for 1~2 hours and then transferred to polyvinylidene difluoride membranes (Millipore) using a wet transfer system at a constant current of 200mA for 1.5 hours. After blocking with 5% bovine serum albumin for 2 h at room temperature, membranes were incubated with primary antibodies of EGR1(CST, #4153, rabbit, 1:1000) or β-actin (protientech, 60008-1-Ig, mouse, 1:5000) overnight at 4 ℃. Membranes were washed and incubated with goat anti-mouse IgG or goat anti-rabbit IgG secondary antibodies (protientech, 1:10000) for 1 h at room temperature. Blots were then developed with the ECL kit (Biodragon) and detected with a chemiluminescence detection system (Syngene). The expression levels of proteins were quantified relative to β-actin expression using ImageJ software (National Institutes of Health).

***Dual-luciferase reporter assay***

Briefly, the 2000 bp sequence of upstream of the transcription initiation site of CDK4 containing potential EGR1-binding sites (-AGTGGGGGTGG-) was constructed into an expression vector (pGL3, Ruibio) containing luciferase to construct the pGL3-CDK4 reporter plasmid. The reporter plasmid can regulate the transcriptional expression of Luciferase. The reporter plasmid was then transfected into the 293T cells by using ExFect Transfection Reagent (Vazyme) according to the manufacturer's instructions. Cells were lysed after different treatments (transfected with EGR1 or EGR1 C>A). The substrate luciferin was added, and luciferase catalyzed luciferin to emit fluorescence (the strongest wavelength was around 560nm). Renilla luciferase's reporter plasmid (pRL-CMV) was used as an internal reference (the strongest wavelength was around 465 nm). Luciferase reporter gene expression was measured with a Dual-Luciferase Reporter Assay Kit (Promega, E1910) and a Centro XS LB960 detector (Berthold). Relative firefly luciferase (RFL) activity was obtained by normalizing firefly luciferase activity against Renilla luciferase activity.

**Supplementary Figures**


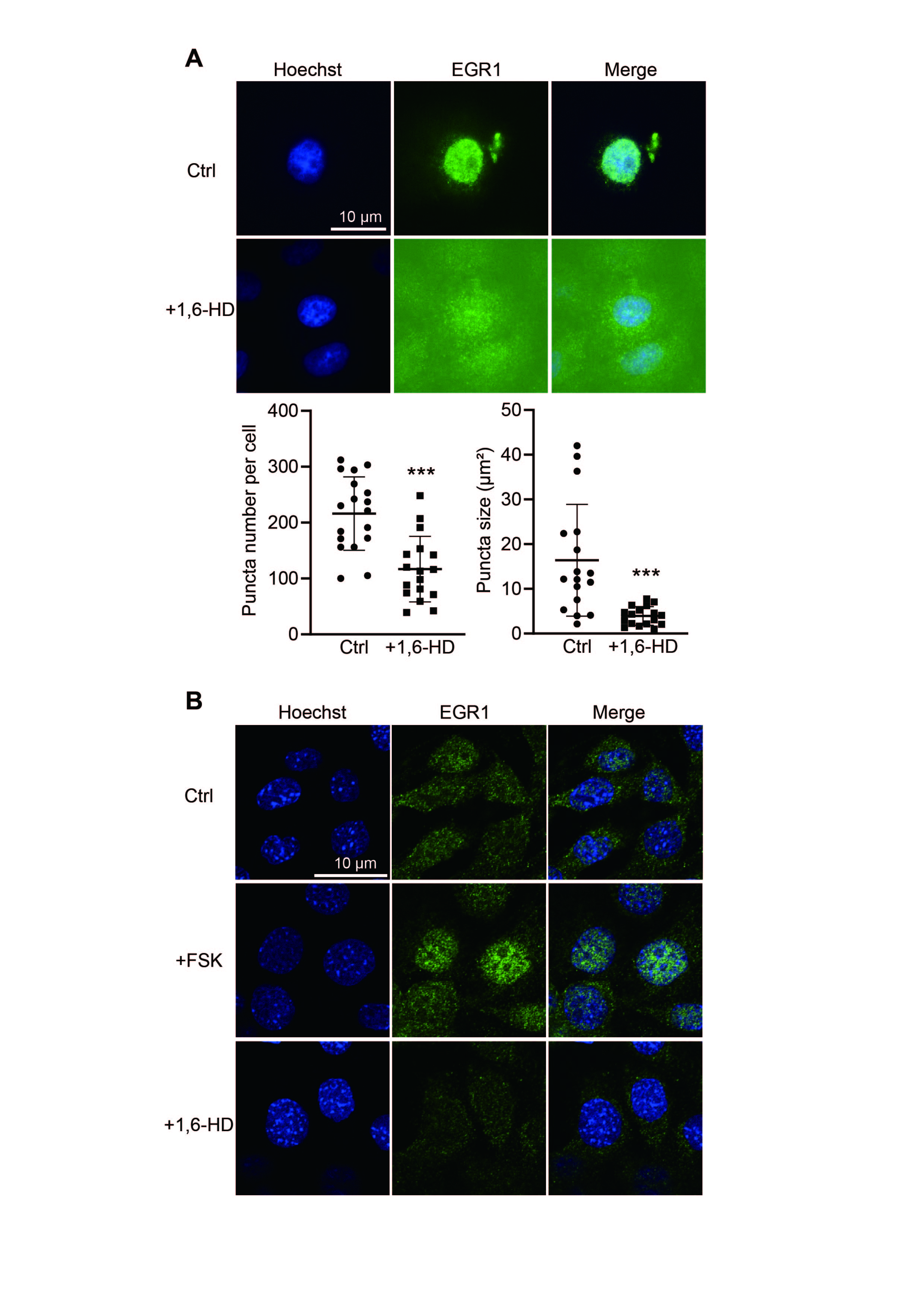


**Supplementary Figure S1. EGR1 nuclear condensates in cyst epithelial cells.**

(A) Immunofluorescence of EGR1 (green) in fixed WT 9-12 cells in presence or absence of 1.5% 1,6-HD (*upper*). Nuclei were stained by Hoechst. Quantification of puncta number and size per cell (*lower*). Bar =10 μm. ****P* < 0.001, *vs* Ctrl group. (B) Representative immunofluorescence images of EGR1 (green) in fixed mIMCD3 cells without or with FSK or 1.5% 1,6-HD. Nuclei were stained by Hoechst. All data are presented as mean values ± SD.


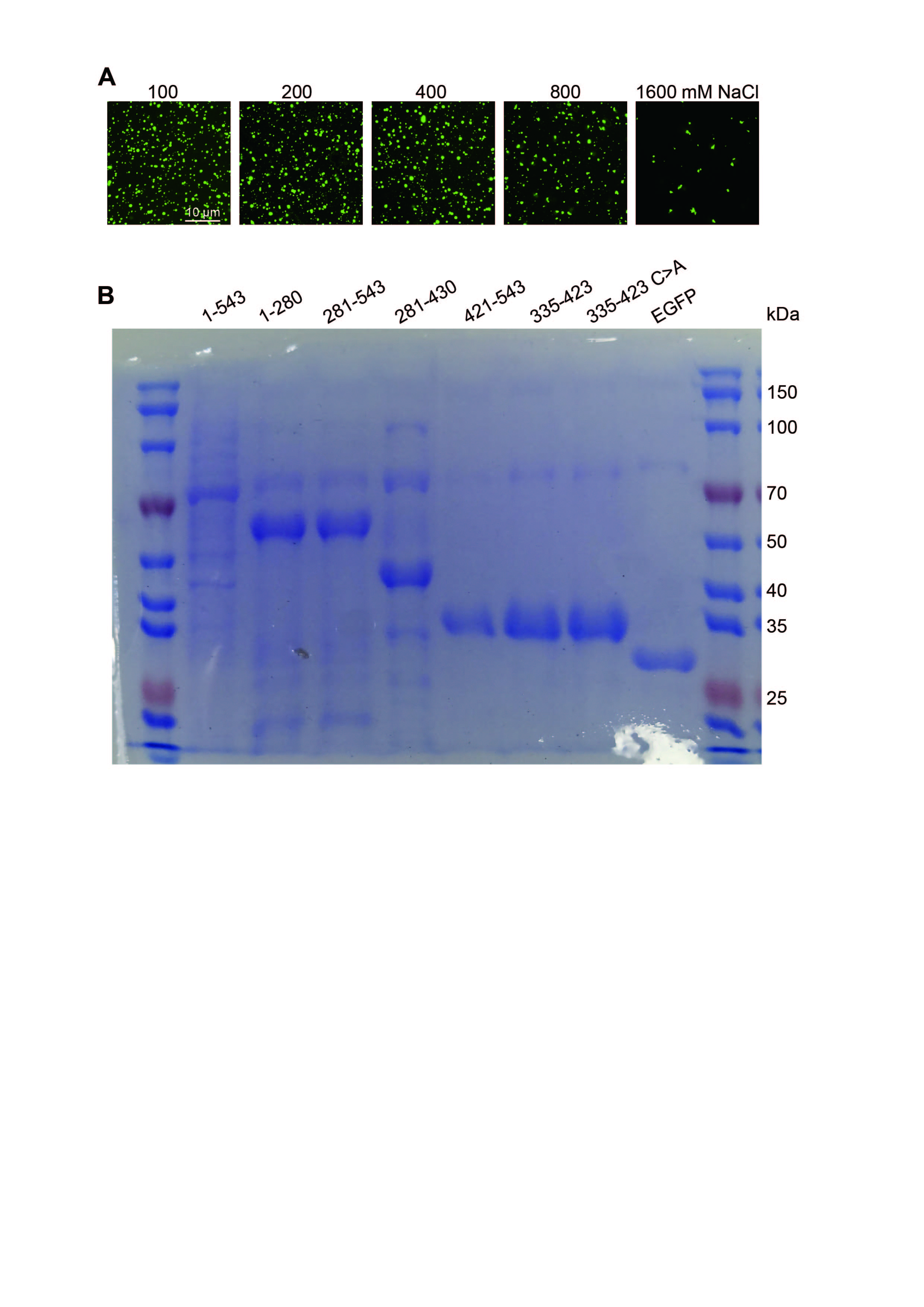


**Supplementary Figure S2. Recombinant EGR1-EGFP truncations.**

(A) Representative images of EGR1-EGFP of droplets in presence of different concentrations of NaCl. (B) SDS-PAGE of EGR1 truncation fusion proteins stained with Coomassie blue.


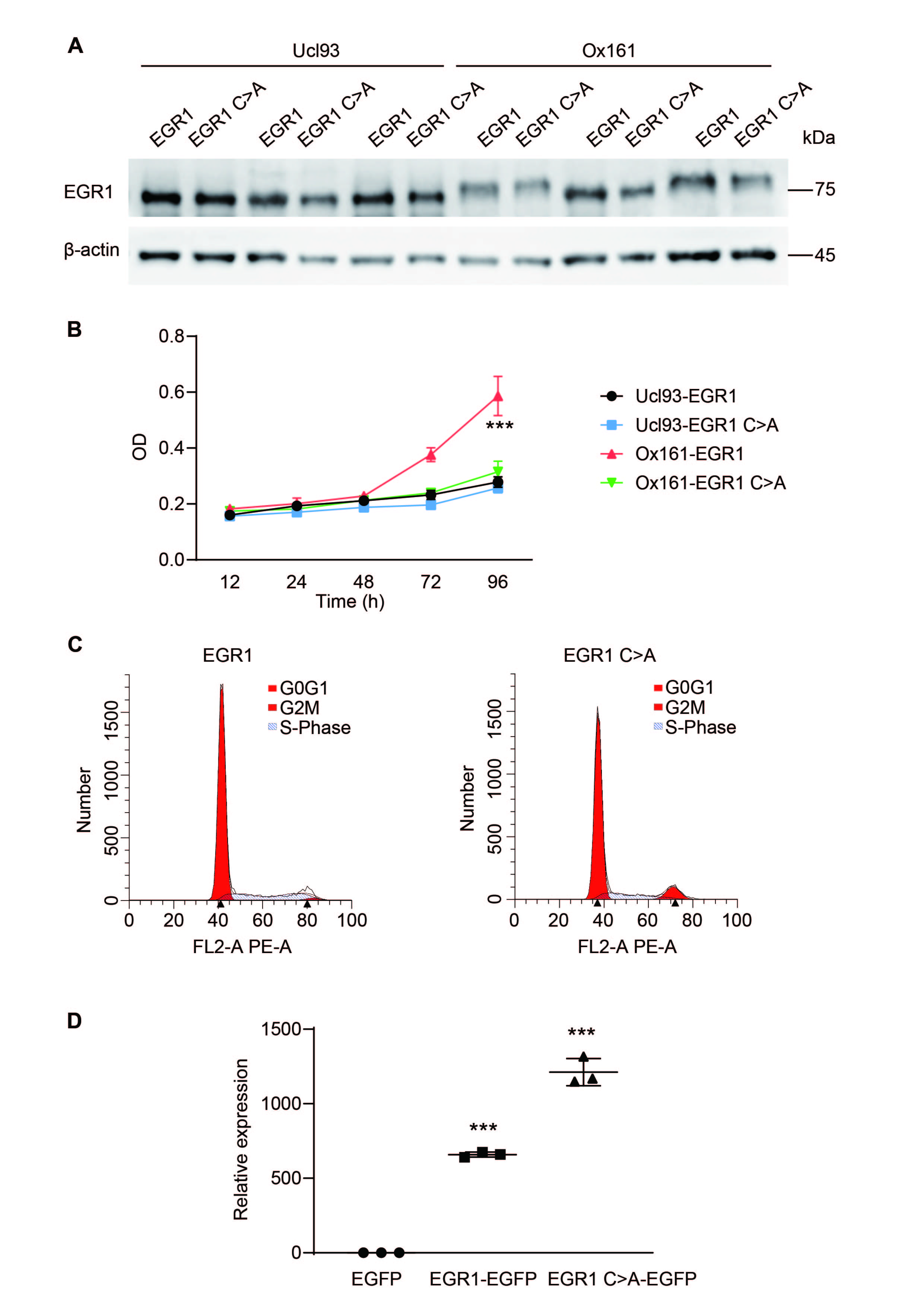


**Supplementary Figure S3. Cells with stable expression of EGR1 or EGR1 C>A.**

(A) Representative Western blots of EGR1 and EGR1 C>A in Ucl93 and Ox161 cells stably expressing EGR1 and EGR1 C>A tagged with EGFP. (B) Cell proliferation was measured by the CCK-8 assay. n=3. ****P* < 0.001 *vs* Ucl93-EGR1 group. (C) Distribution of cell cycle in Ucl93 cells stably expressing EGR1 and EGR1 C>A tagged with EGFP, which was assessed by flow cytometry. (D) RT-qPCR analysis of mRNAs for MDCK cells stably expressing EGR1 and EGR1 C>A tagged with EGFP. ****P* < 0.001 *vs* EGFP group. All data are presented as mean values ± SD.


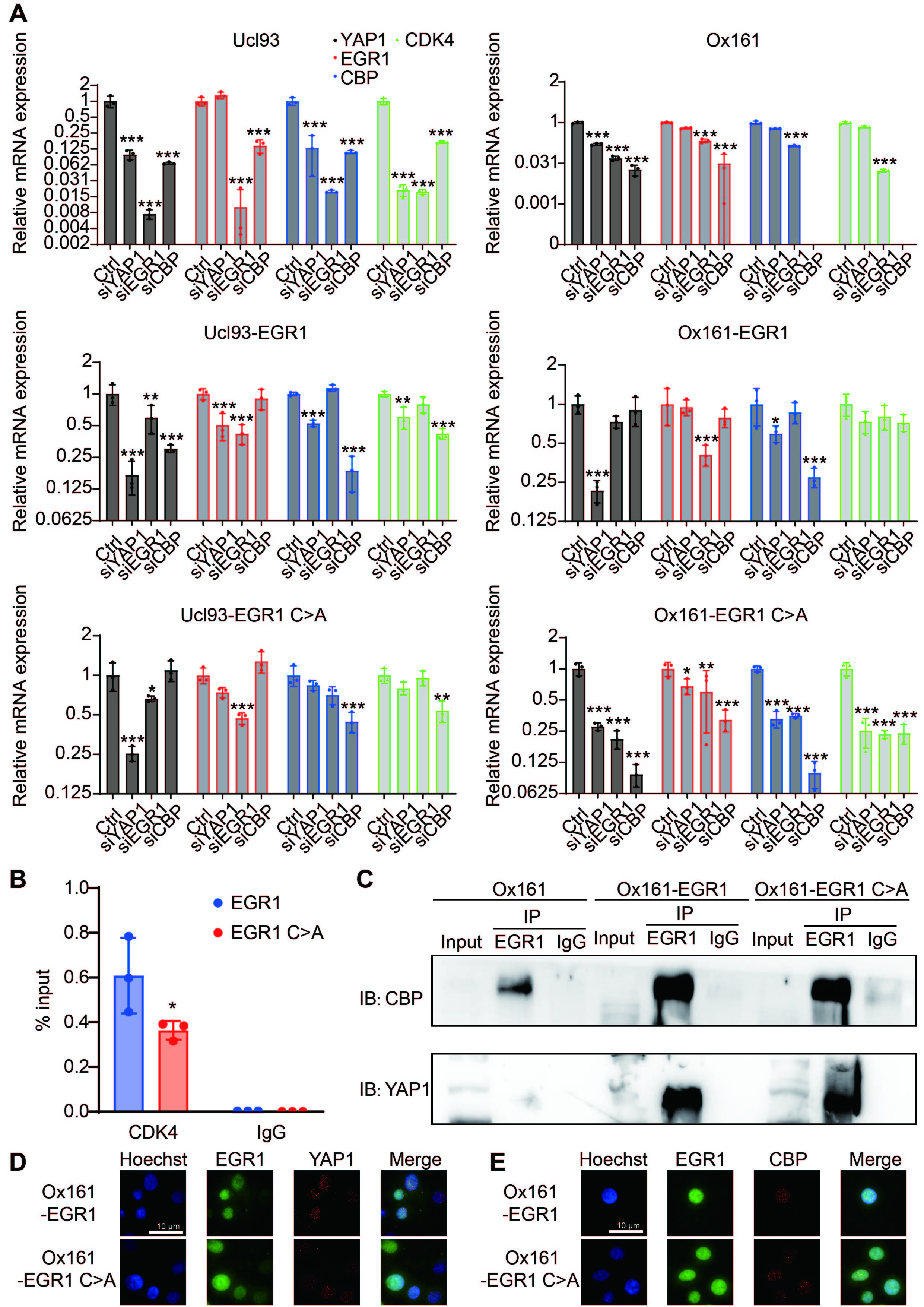


**Supplementary Figure S4. EGR1 phase separation with YAP1 and CBP.**

(A) RT-qPCR analysis of mRNAs for cells stably expressing EGR1 and EGR1 C>A and transfected with siRNA. ****P* < 0.001 *vs* control group. (B) ChIP-PCR analysis of EGR1 and EGR1 C>A binding to CDK4 promoter. n=3. ****P* < 0.001 *vs* EGR1 group. (C) Co-IP assay testing the interaction between EGR1 and YAP1 or CBP. (D) Immunofluorescence of the co-localization of EGR1 and YAP1 in Ox161 cells stably expressing EGR1 and EGR1 C>A. Bar = 10 μm. (E) Immunofluorescence of the co-localization of EGR1 and CBP in Ox161 cells stably expressing EGR1 and EGR1 C>A. Bar = 10 μm. All data are presented as mean values ± SD.

**Supplementary Table**

**Supplementary Table S**1. Primers for RT-qPCR

|  | Forward primer 5’-3’ | Reverse primer 3’-5’ |
| --- | --- | --- |
| CCND1 | ACCTGGATGCTGGAGGTCTG | CAGGGGGATGGTCTCCTTCA |
| CCNE1 | ATACTTGCTGCTTCGGCCTT | TCAGTTTTGAGCTCCCCGTC |
| CDK4 | TTGCGGCCTGTGTCTATGG | GATCAAGGGAGACCCTCACG |
| CDK6 | ATAAAGTTCCAGAGCCTGGAG | CGATGCACTACTCGGTGTGAA |
| β-catenin | ATGATGGTCTGCCAAGTGGG | GGCCATCTCTGCTTCTTGGT |
| actin | CACTCTTCCAGCCTTCCTTC | GTACAGGTCTTTGCGGATGT |
| CDK4 promoter | GGGCAAGGTATGGATGTGGT | GGACATGTGGAGTGTTGGCT |
